# CSR–brand relationship, brand positioning, and investment risks driven towards climate change mitigation and next perspectives emerging from: “Litigation, projections, pathway, and models”

**DOI:** 10.1007/s43546-022-00374-4

**Published:** 2022-12-20

**Authors:** Olukorede Adewole

**Affiliations:** Literary Edifice, Rome, Italy

**Keywords:** Brand positioning, Brand relationship and CSR, CSR, Climate change mitigation and litigation, Environmental sustainability, Investment risks, Models

## Abstract

This study delineates the relationship between CSR and brands, unveiling pragmatic steps towards achieving the sustainable business environment, while unveiling its potential towards climate changes mitigation cognizant of investment risks, leading to an action plan-framework for proffering practical solutions coupled with establishing future paths and projection towards addressing climatic changes consequences, risen incidences in litigation trends and environmental issues. Trends manifesting in risen litigation incidences linked to industrial and economic activities that adversely impact the climate, environment and society makes it imperative to look extensively beyond prediction models while establishing and building on brand relationship with CSR, while strategically establishing a practically realistic business model for translating this relationship to value-creation and applied in abating climate change, addressing all environmental concerns, redressing litigation incidences, among other issues resulting from impacts of business and socio-economic pursuits of humans. The twenty-first century realities towards a green planet demands doing business strategically, optimize resources by imbibing investment risks as a trend and organizational culture—strategic fit, adopting brand as a potential tool for addressing climate change and environmentally related activities and adversaries from business activities and negligent practices from such, while achieving climate change mitigation as outlined and extensively inundated.

**Supplementary Information:**

The online version contains supplementary material available at 10.1007/s43546-022-00374-4.

## Introduction

Obviously, business and economic activities and human endeavors in the social, economic, and industrial spheres have done and brought great and tremendous impacts on the environment.

A number of issues have recently emerged and remained subjects of crucial debates and arguments among academics, policy and decision makings, political to action or protest groups, and NGOs from the industrial, economic, climate changes, sustainable marketing, and business.

Some of these issues and consequences include; climate changes, environmental hazards, and related causes from negligent practices from corporations, companies, and organizations and rising incidences of jury cases and litigations brought against corporations from impacts if their activities, activism, and protests against the state as subsequently outlined.

These issues constantly evolve around business, environment, CSR, climate changes, and sustainability from over 30 decades till present realities evidently seen and known manifestations.

- Armstrong and Kotler (2008) reported that socially responsible marketing is in the best interest and benefit of society, also evident as outlined (Kotler and Lee 2004; Kotler et al. 2018).
- Human socio-economic activities and businesses have drastically impacted the environment manifesting to climate changes.

It is essential, quite pertinent, and crucial to note that, as corporate social responsibility (CSR) continues to gain place, prominence and attraction or interests, and keen attention in management circles, its translation into actual managerial practices and performances remains a challenge or tough task for many organizations (Carroll and Buchholtz 2011; Jamali and Neville 2011; Aguinis and Glavas 2012). In fact, it might seem confusing if not decisive enough on what part or component of CSR to pursue in its multidimensionality, as it consists of 3–4 key strands from “economy, ethics, philanthropic and legal” aspects, and various variants that have emerged.

CSR can be inferred to be key, salient, and significant to any key positions, strategies, and implementation in present-day business, and an essential component of the business model.

Whatever the picture might seem to be, tough tasks or challenges, a company has to be decisive, and pursue a clear strategy, and carve and design an appropriate strategy and social responsibility approach in a corporate sense. The brand perspectives of CSR have been the pivot and center piece of this discussion as outlined, and how it can translate to impactful meanings and benefits on the environment, climate change mitigation, risks aversion, redressing incidences of rising litigations, etc. as enumerated.

We are conscious of one clear and unprecedented fact, the vast and enormous changes technologically and economically seen and brought about by drastic revolutions industrially, massive industrialization, innovations, and extant dynamics over a span of over 3–4 decades ago have drastically impacted the environment culminating in climate changes and the number of environmental-related issues associated and connected with man socio-economic trajectories and changes seen in dynamics.

This study and investigation would address business impacts and activities of corporations, businesses, firms, and organizations on the environment while proffering practical and potential steps to address major trending issues arising and emerging from business and marketing practices on the environment and societies at large.

One major trajectory and new perspective clearly drawn and emphasized here is the prospect of imbibing investment risks into the business model, and organizational culture, while examining the concept of a brand with CSR and exploring its potential in combatting and addressing climate changes within a strategic view.

The consumers’ motivation for CSR and their perception of corporate brands (Mody et al. 2017a, b), and consumer satisfaction (Yang et al. 2017) are important factors affecting consumer loyalty (Yang and Yin 2019). Companies can employ social media to actively spread the appropriate information of the brand image to consumers (Cheung et al. 2019), and to significantly influence the consumers’ perception of the brand agreement with them by conveying the consistency of brand image.

Strategically for corporations and organizations or businesses at large, by emphasizing on brand, realizing the potential for brand translation to equity through CSR, imbibing investment risks and seeing as a culture in the organizational context, and building a model based on these attributes, such a business model can be a potential and significant tool towards resource optimization and checking against wasteful use of resources and a hedge against litigation redress seen and often brought against corporations, entities and institutions or governments as subsequently highlighted and enumerated.

While CSR discourse has extensively revolved around; “society, economic and philanthropy” as reflected in works of literature and fundamentally seen going further into proactive ways of mitigating climate change and environmentally related issues incorporating investment risks and a culture entrenched around thus while looking beyond climate change and emission gases predicting models conscious and cognizant of rising litigation cases becomes highly imperative.

Investment risks like insurance or hedge are a pool and proactive tool or measures and within a cultural and strategic context that can be pragmatically imbibed and adopted by corporations against climate change as explicitly presented here and discussed.

Outline of the article, and paper presentation in the realization of existing literature gaps and raising new frontiers for investigation

- This paper sought to address climate change by working towards mitigation by incorporating CSR into the brands while unveiling the conceptualization of investment risks and brand positioning while making future templates towards climate change and prediction models conscious of frequently emerging climate changes challenges and rising incidences of litigations
- It is sought to provide pragmatic steps and approaches to be taken in the present realities seen
- This paper emphasizes the essence, benefits, and significance of incorporating climate and investments risks into the business model
- New propositions, hypothesis frame, emerging trends or projections, and novel models are carved and presented ending with salient recommendations

This study is based on explicit literature and in-depth narrative explanation of real, existing, and emerging facts; extensively delineating conceptual framework, theories, and novel structures within hypothesis frames, theories, and formulations while extending to a comprehensive and detailed elaborated quantitative treatment statistically based on data acquired and a poll done based on random sampling and questionnaire administration to respondents drawn from some firms and public domain in Rome, neighborhood and proximity capturing up to a minimum of 225 responses which was subsequently analyzed.

A normal distribution or approximately ‘Gaussian is an expression of the form:

$$y=\frac{1}{\sqrt{2\pi {\sigma }^{2}}}{e}^{-\frac{{(x-\mu )}^{2}}{2{\sigma }^{2}}}.$$

with sigma: *σ* denoting the standard deviation for a distribution set with mean: *μ*; a standard number or Gaussian with mean: *μ* = 0 and standard deviation: *σ* = 1 would be expressed as:

$$y=\frac{1}{\sqrt{2\pi }}{e}^{\frac{{x}^{2}}{2}}.$$

The validity of the assumption of normal distribution was subsequently verified and tested for the data set as subsequently shown and obtained from the kurtosis and skewness.

The skewness and kurtosis in principle fundamentally and basically explain the tendency for data to be skewed left or right tail.

By the rule of thumb, for a large population group, a minimum sample size of 30 is okay; however, in this case, a sample size of 225 was applied overall.

Finally, as we try to alert and raise consciousness or awareness of the consequences of activities impacting climate change and seek efforts towards practically realistic mitigation steps such as the incorporation of investment risks as culture attributable to the brand or corporate structure in the context of CSR, brand relationship and positioning as extensively delineated in this presentation, we need to ask and answer the pertinent question as follows.

Inferring and suggesting from the literature, it is essential to say that it is not possible for a brand to have no brand equity aligning with Keller (1993) who opined and asserted that any potential encounter with a brand can influence its mental representation kind of information manifested in the memory of the consumers. The framework applies the concept of appropriable value to the brand equity literature in consistency with the combinations of literature on mergers and acquisitions (Barney 1986; Ailawadi et al. 2003) and current managerial practice seen in P&G value pricing (Keller and Lehmann 2002; Krishnan 1996).

In fact; CSR can enhance the brand relationship, facilitating and building brand loyalty and in turn promoting and lifting the brand image;

In line with (Jia 2019); brand image to a certain extent can affect and determine the direction of market development which can affect and significantly influence the purchasing willingness and identification of the consumers towards the product.

It is pertinent to say and identify that brand image can be enhanced by consumer perception of ethics (Ahmed et al. 2020; Iglesias et al. 2019), consistency of advertisement and promotions (Arbouw et al. 2019); further more Li et al. (2017) and Lu et al. (2020) brand reputation and consumer trust may create an overall positive impact on the corporate image.

In a further revelation, CSR performance of a company could trigger or bring higher stock returns (Lins et al. 2017). This can in turn enhance and favorably build the brand's reputation (Asmussen and Fosfuri 2019).

Negligent of responsible actions towards climate change mitigation and ignoring investment risks would come with and manifests in consequences that could severely impact the finances of organizations, companies, and corporations in pursuing redress against litigations and actions sought against such bodies by plaintiffs.

Litigation, projections, CSR, pathway, and climate change models, where are we?

While the climate change models and predictive tools could be helpful in gaining possible insights and grasping ideas into future trends, we have to look beyond the models embracing more pragmatic and realistic actions and tools more responsible by imbibing investment risk culture tied and built around the corporate brands and strongly emphasize corporate social responsibility and other tools among “stakeholder’s engagement, social contract theory, and societal marketing”, essentially adopting CSR as a key component of the business model as a potential tool, means, and device for abating and mitigating climate changes, redressing rising incidences of jury cases and litigations, activism and actions or protests against companies, states, institutions and organizations, which could be militating against the smooth functioning and painting bad pictures or perceptions of images or the brand as subsequently presented and explained.

These steps would drastically trigger, thereby enabling and engendering brand relational building, promoting corporate esteem, image and geared towards creating customer value, socially and environmentally responsible, and establishing a sustainable future beyond the present and profit motives.

### Research questions

Borne out of need and in line with the main purpose of this study to establish the relationship between CSR, and brand while exploring the potentials of CSR as a strategic tool, viral component of the business model and addressing key environmental issues, while corporations can benefit as well by the adoption of investment risks, optimized uses of resources, the following research questions are presented:

1. Is there a relationship between brand and CSR roles of organizations?
2. What are the role and potentials of the brand and CSR in mitigating climate change risks?
3. How do individuals and entities within a corporate organization perceive the brand?
4. Is there a link between the brand, CSR and investment risks?

## Literature

CSR, environment, climate change, and sustainability have become and emerged as crucial hot topics or concepts for debates, arguments, and discourse in academia and industrial domains.

A number of various schools of thoughts and business scholars have put forth, and pushed various arguments, thoughts or opinions tenaciously in explaining the relationship between sustainability and CSR.

It becomes imperative in the present and emerging realities to extend the brand concept as mentioned and introduced earlier to fuse and mesh investment risks in considering the brand relationship while we try to seek more pragmatic and practical ways of mitigating climate change and related environmental issues and adopting the concept of CSR as a veritable tool for building a strong brand relationship by way of value creation and consumer intimacy; again in as much as various strands of CSR have emerged, this becomes expedite and very interesting to dwell more on branding in connection with CSR, and explore its potentials.

As identified by Sethi (1973); a legitimacy gap exists when an organization does not meet or cannot deliver the expectations of society and their wants which can put in jeopardized position the organization’s positioning or a threat such as losing clients, customers, or donors; attract government sanctions or penalties, arouse and stir citizen protests and difficulty in attracting employees and could impact on talent attractions hazardously.

Cox (2013) recognized and described the 1970s as a period representing the passing of multiple landmark environmental laws in the U.S. stipulating and requiring polluting companies to take hold and be responsible for some of their operations that caused externalized consequences in the past.

Suchman (1995, p. 174) described legitimacy as a generalized perception, assumption, or opinion that construes the actions of an entity as desirable, proper, or appropriate within some socially built system of norms, values, beliefs, and definitions tied and built around various relevant social and stakeholder theories.

In the thought and opinions of Berkhout (2005), CSR plays a vital role in promoting and enhancing sustainability. By embracing and adopting the principles of CSR, a firm is conscious or cognizant of how it utilizes its resources, strives towards efficiency, and optimizes at the same time reducing negative impacts and severities not only to the environment but also to the economy and the society as a whole.

Asking this pertinent question in line and bearing with this study, why not incorporate climate change and investment risks?

As highlighted and most cognizant and significant for Carroll (2008), the most relevant societal concerns and expectations of corporate behavior during the 1980s revolved and lying around “environmental pollution, employment discrimination, consumer abuses, employee health and safety, quality of work life, deterioration of urban life, and questionable/abusiveness practices of multinational corporations” (p. 36).

As mentioned earlier a number of business scholars and academics from diverse and various Schools of thought have put forth various arguments in explaining the relationship between sustainability and CSR.

According to Berkhout (2005), CSR plays a vital or key role in promoting and enhancing sustainability as mentioned earlier.

CSR gives adequate room and place for full stakeholder participation not ignoring any party and ensures the community and dwellers in the locations where companies operate are carried along and philanthropically give back to the communities beyond economic gains and own profits or solely company’s interest by being socially responsible and participating in major communal projects to foster development and overall social well-being.

Porter and Kramer (2006) advanced and put forward a different model for strategic CSR that capitalizes on leveraging the unique resources and competence of the firm both internally and matching those externally to the needs of the situational context or expectation and manifestations seen.

This strand or segment of strategic CSR can be integral to a company’s profitability and competitive positioning presented by the authors. Their proposed context-focused strategic CSR approach requires firms to use their unique attributes to address social needs in the corporate context so as to achieve convergence and establish a link between social and economic goals. It was also reiterated as they differentiate strategic CSR from responsive CSR with the former going beyond attuning to evolving stakeholder concerns and mitigating adverse effects of corporate activities to carve out a distinctive and competitive niche for the firm.

Armstrong and Kotler (2008) reported that socially responsible marketing is in the best interest and benefit of society. This is also supported as highlighted (Kotler and Lee 2004; Kotler et al. 2018) Based on this fact, thus it is extremely essential in the recent marketing and business environment in the short-term and long-run.

CSR as a business model helps companies to self-regulate or control activities that impact stakeholders, including the general public (Ferrell and Hartline 2011). These activities could be seen manifesting and revolving around climate changes, environmental related issues and how these impact the stakeholders and prompting some responses and reactions against corporations that bring these impacts as seen in rising litigation trends as trending instances where communities and parties affected now seek legal redress and demands for compensations.

Besides economic implications and considerations, marketing strategies significantly reflect on and impact the prevalent social value system and environment.

The Interrogative and investigative Panel on Climate Change (IPPC, 2014) released a report tagged Climate Change and consistent with the document, UNFCCC (2013): “The Physical Science Basis; an explicit report which describes the very high probability and tendency by the late 21st century of risen temperature and more heat waves generation over most land areas; increased volumes of precipitation, frequency and intensities, elongated duration of drought; elevated or risen and intense cyclone activity, extreme high sea level rise, turbulences and surges”.

As Carroll (2015) pointed out, exemplified, and explained, during the 1990s, the globalization process and trend increased the operations of multinational corporations which now faced diverse business environments abroad, while some of them are noted and characterized by weak regulatory frameworks and templates with no adequate background to cope with emerging realities.

For these global corporations, it meant new opportunities that came along or resulted from rising global competition and striving for new markets, increased reputational risk due to a growth in global visibility, and conflicting pressures, demands, and expectations from the home and the host countries (Carroll 2015) were evidently seen in the scenes.

Re-iterating; CSR is in the best interest not only of the society but also the firm that practices it; its potential benefits are numerous and huge including increased and enhanced brand reputation, brand loyalty or image, and profit maximization.

A firm that practices CSR believes that its profit is enhanced and can achieve profit maximization with long-term goals and benefits to overall sustainability. In this bearing, it has been reported that firms that practices CSR believe their revenues have increased over time and eventually proved helpful for the long-term profit maximization of the firm (Lu et al. 2019).

Doing business sustainably or in a sustainable manner is quite crucial, key, and highly essential in the present state and realities!

It is pertinent and highly essential to devise the right strategies and move in a strategic direction toward managing and handling the present situations and emerging realities associated with climate change and how doing business has exerted an impact.

It is quite and highly imperative to note that various companies have taken different approaches or policies to sustainability in their CSR models which can be considered and extended along the strategic fit.

It is interesting to note, and know that the most common or usual approach is incorporating the principles of CSR and environmental sustainability in their business models or strategies, and as such or an instance and example taking into consideration the environmental and social repercussions or impact of their business activities.

Besides corporate image it has become evident from emerging facts and studies that consumers are more attracted to companies and firms that are socially responsible in look or appearances (Irshad et al. 2017); this is an image that builds more brand reputation, relationship, and can improve consumer behavior and purchase intentions for a particular brand.

Local communities have the paramount and key roles or responsibility to determine the particular elements or components to sustain, what to expense and how to expense them. This is not only because these decisions directly impact them but because they have crucial and significant roles to play in the processes that aim at enhancing responsibility (Berkhout 2005) or modifying and shaping such.

Lu et al. (2019) identified CSR as an integral part of a company’s activity in building trust among consumers. CSR can enhance the brand from trust and reputation, in fact, Mahmood and Bashir (2020) established that brand reputation can be translated to brand equity. This will, in turn, be of huge advantage to the company which can gain the mutual trust, confidence, and loyalty of its consumers and customers.

Ingram et al. (2013) stressed and emphasized that increased extreme events and climatic variability are projected to increase. This is a bearing and basis in line with the perspective of this study and research for the unveiling and examining climatic change models and projections among those of greenhouse and atmospheric gas emissions as subsequently demonstrated in this presentation while also looking vividly into their consequences as mentioned earlier an instance leading to rising incidences in litigations and pending jury cases brought against corporations, firms, companies, organizations and governments for their negligent and adverse or damaging alleged roles sin climate changes, causes, environmental impacts, and related events.

As projected and asserted by 2050 and pointed – out (Dow, Berkhout & Preston, 2013); the economic impact of extreme events and climatic variability will increase financial losses by trigger up to 3.9 times those presently experienced.

As highlighted and explicitly discussed so far above, industries and businesses seem to capture and have a sense of the essence and idea of corporate social responsibility, CSR, and a sense of doing business in a socially responsible manner; why some businesses, institutions, and industries or corporations are stemming towards CSR and imbibing into their activities, operational modules and businesses scope much as possible and to a considerable extent, the question still remains are they conscious of climate change risk, to what extent is the prioritization towards such a possibility of climate change and investment risks reflecting in their operation scope, business models and strategies?

This looks and points to a gap for further literature inclusion, investigations, and pursuing a deeper and clearer perspective for future business models to emerge in the present realities seen so far and the extant business environments where climate has been significantly and drastically impacted by business activities, and furthermore the rising incidences of litigation suits and pending cases hanging in courtyards and jury desks for hearing, calling and ratification or pronouncements.

Seeing investment risks adoption as a strategy and extension of the organization’s strategic fit:

Thus in the line of this context(s), presentation and directions investment risks and climate change risks can be portrayed, presented, and seen as a strategy and in-line of extension along the strategic fit carved into the business model, plans, and vision of the company, organization and corporate entity.

Furthermore, and subsequently, Porter and Kramer (2011) presented the “Creating Shared Value (CSV) framework linking strategic CSR and sustainable outcomes” to a deeper appreciation of societal needs and a better understanding of the true bases of company productivity as given and explicitly presented.

Pertinently, and significantly, strategic CSR is becoming of increased importance and has been emphasized and outlined in literature (Heikkurinen 2018).

Heikkurinen (2018) highlighted and emphasized the need for companies to be philanthropic and look beyond their profit interests and goals while taking care of the natural environment.

Strategic CSR as highlighted, enumerated, and pointed out significantly entails formulating CSR strategies that are aligned with the strategy of the firm and can generate both short-term and long-term outcome values (Porter and Kramer 2011).

CSR is an act driven towards balancing between three related areas of business, that is, economy, society, and environment (Aleksić et al. 2020; Berber et al. 2019). CSR includes the elements of a circular economy (Vukadinović and Ješić 2019), which is seen as a sustainable concept for the development of the economy.

While the literature has examined the relationship between CSR and brand and the prospects or idea of translating brand reputation to brand equity, for instance, Mahmood and Bashir (2020), the roles and potentials of adopting brand to abate climate changes and mitigation and addressing key environmental issues and rising activism against states from protests and jury cases or litigations brought against corporations and companies on their acts that impact on climate and environments or issues of environmental concerns can be addressed considerably as explored in this study and unveiled.

We do obviously realize that 'CSR, environment, climate changes, and sustainability have become and emerged crucial and hot topics or concepts for debates, arguments, and discourse in academia and industrial domains.

It has become highly imperative in the present and emerging realities to extend the brand concept as mentioned and introduced earlier to fuse and mesh investment risks in considering the brand relationship while we try to seek more pragmatic and practical ways of mitigating climate change and related environmental issues and adopting the concept of CSR as a veritable tool for building a strong brand relationship by way of value creation and consumer intimacy. Furthermore, this can fill create a gap and existing between business, company or corporation, and the society with the environment.

Finally, as buttressed and supported by the framework of this study, which transforms and translates brand into equity, emphasizing the society and the need for investment risk, subsequently as presented into the extended and working framework for this study and within the identified goal and gaps beyond extant and existing literature is to extend the ‘stakeholder aspect into brand translation and transform into equity, while addressing key environmental and climate change issues as well as concerns of rising incidences of litigation as mentioned earlier.

### Societal marketing

**Three key considerations of societal marketing**

**Fig. 1:**
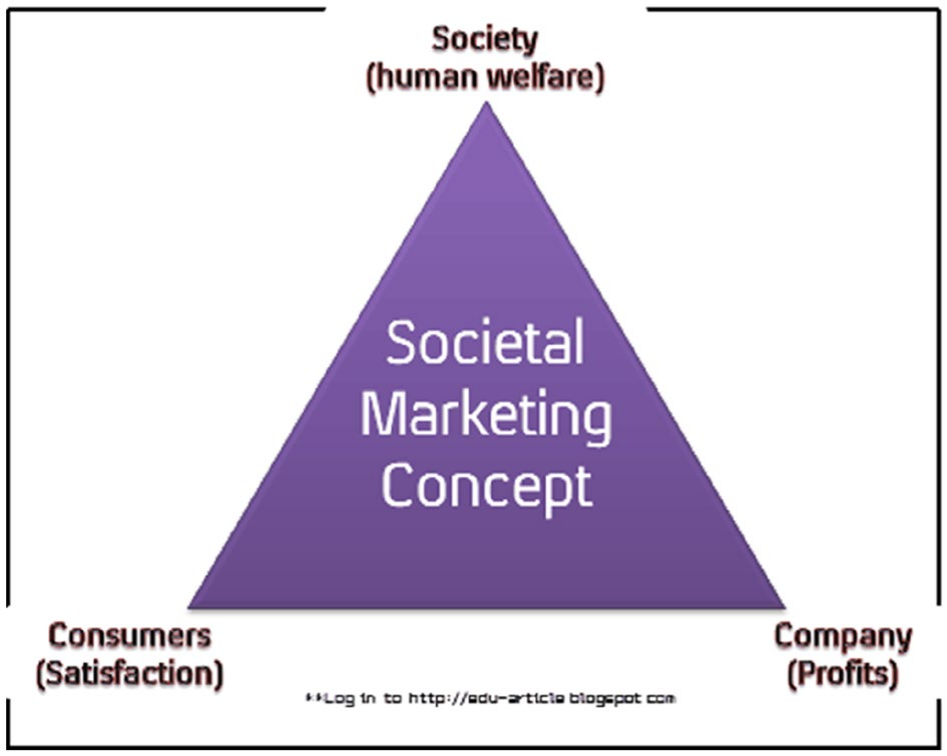
Chart showing societal marketing key considerations; Source: http://socio-articlebloggspot.com

Societal marketing can strongly enhance sustainable marketing and engender a long-term goal, vision, and sustainability (Fig. 1).

Furthermore, societal marketing can be linked and associated with CSR as it considers society through well fare consideration and human well fares beyond the company’s profit goal.

It can be inferred; society marketing is an all-encompassing frame within and buttressing or underlying the ‘triple bottom line.

### Stakeholders and social contact

Stakeholder’s collaboration and engagement are key underlying features and characteristics of CSR.

Investment risks in the light of this, that is from the societal perspective and stakeholder’s collaboration implies the company considers the need and well-being of the society caring and concerned to pull and save incentives towards resource optimization while averting environmental dangers and hazards as much as possible from their activities.

Allen (2016) pointed and identified crave for or quest to be perceived and seen as a legitimate corporate actor significantly justifies the driving force behind CSR and sustainability efforts and strives.

Milton Friedman’s (1971) now classic essay appeared to a receptive business community.

Friedman’s was of the position and stance that the social responsibility of business is to make profits.

Cheney et al. (2007) presented that decision makings toward scarce resource allocation should be the sole responsibility of the political echelon and not the market.

Friedman opined that entire parties concerned and all would benefit if businesses make and base decisions on shareholder value.

This was entrenched and imbibed characteristic of the neoliberal philosophy which came into force and declaration through the instrumental orientations of the trickle-down economics perspective which led to a ‘Discourse till now which gained dominance and strength during the Regan eras in the US.

As highlighted in a previous study, firms have several stakeholders who compete for organizational resources; hence borne out of this fact or emerging reality, there is an expedient need for firms to identify strategies or ways for managing stakeholders (Bryson 2005; Michelon et al. 2013).

The type of stakeholders proactively engaged and resources control strategy or measures adopted impact significantly on the firm’s corporate strategy. From a business-driven viewpoint, perspective or orientation, stakeholder theory interest covers three premises: that organizations have stakeholders who impact their activities; these interactions impact specific stakeholders and the organization, and perceptions of major stakeholders impact the viability of organizational strategic options (Simmons 2004) and alternatives.

### ‘Precepts, conceptualization resident in theory and fundamental layouts:

#### Theoretical framework outline

**Theoretical and contextual framework**

**Fig. 2:**
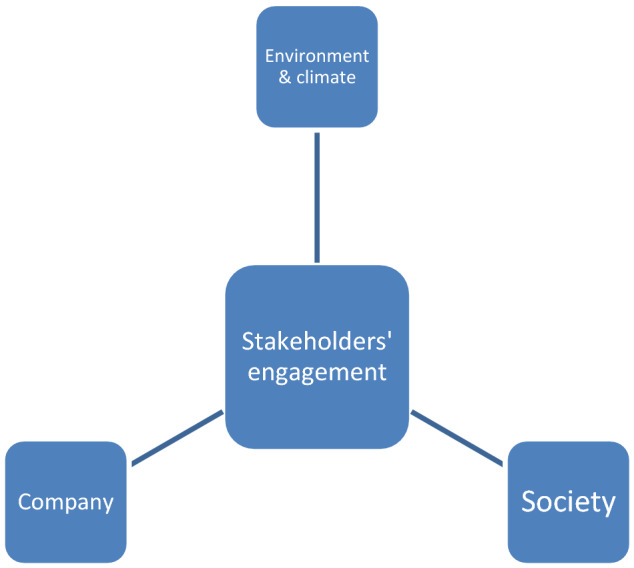
Contextual and theoretical frameworks for CSR built on stakeholder’s engagement; source: Author’s draft and present study. Triple bottom line

The theoretical framework of this study is based on the stakeholder’s concept and engagement as presented and illustrated driven around the “environment, company and society” as primary and key stakeholders (Fig. 2).

Following from the basis and fundamental precepts built and entrenched around “society, economic and environment”; it is highly imperative, crucial, and exigently pertinent to link and associate CSR, brand, and examine the potential benefits of climate change mitigation for the good of businesses, society, and environment in moving into a new paradigm, era and shifts that ensure sustainability.

**Fig. 3:**
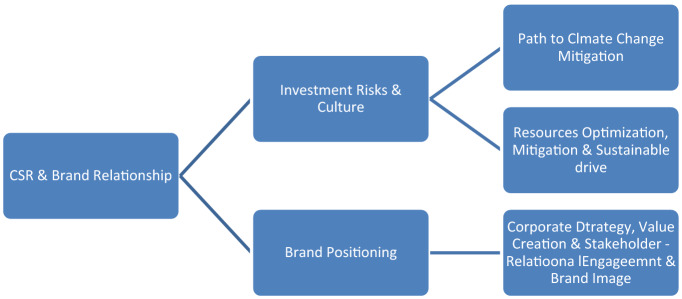
Working and extended frame for the present study and basis for hypothesis formulation and theory. Source: ‘Author’s draft presentation and present study: 2021

**Fig. 4:**
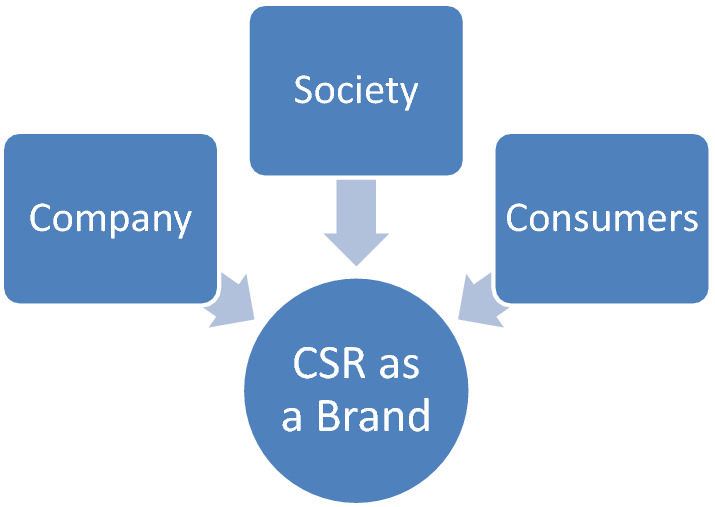
CSR as central and key to the company, consumers, and society; source: present study draft and author’s draft

The triple bottom line, which comprises; “business, society and environment or climate” can be extended to merge CSR and branding in my observation and proposition as presented in Sect. 2.2 below from Figs. 3 and 4.

The above framework transforms and translates from the ‘stakeholder’s aspect and context is then subsequently presented into the extended and working framework for this study from the ‘stakeholder’s aspect, engagement, and the triple bottom line, and within the identified goal and gaps to extend the ‘stakeholder aspect into brand translation and transform into equity, while addressing key environmental and climate change issues as well as concerns of rising incidences of litigation as mentioned earlier.

### Working frame: template and frame structure for hypothesis and theory

This is the proposed working frame and template structure for this research investigation on which the hypothesis frame and theory resides and built upon mentioning and extensively discussing and culminating in the social contract theory and societal marketing principles, risen litigation incidences, climate change, and projection models all-encompassing as subsequently reflected and extensively elaborated all through the entire length, frame and structured layout of this work and research.

In this light and revelation; the following model emanated from this study as presented:

CSR goes simultaneously, or hand in hand, and is strongly associated with a smart brand strategy.

The implication and impact of this template, framework, and structure are that beyond the profit goals of the business or company, from resources optimization, there is a keen interest in protecting the environment by conceiving the brand as a key nexus in ties and connection with the culture, close association with the people or consumers, and developing zeal to pursue environmental interests that could safe and protect the climate following mitigation, invariably, and in turn, this is a potential and strategic step in mitigations, dressing down and checking incidences of rising litigations and jury cases against corporations, states or institutions, etc.

#### Formulations

##### Hypothesis

1. By incorporating investment risks and climate change risks; corporations can achieve optimal utilization of resources.
2. Investment risks should be seen and perceived as a culture, style and manner embedded into the CSR, brand, corporate structure, and strategy of an organization.
3. A hybrid novel business model and sustainable marketing template as a working frame can be built meshing investment and climate change risks.

**Fig. 5:**
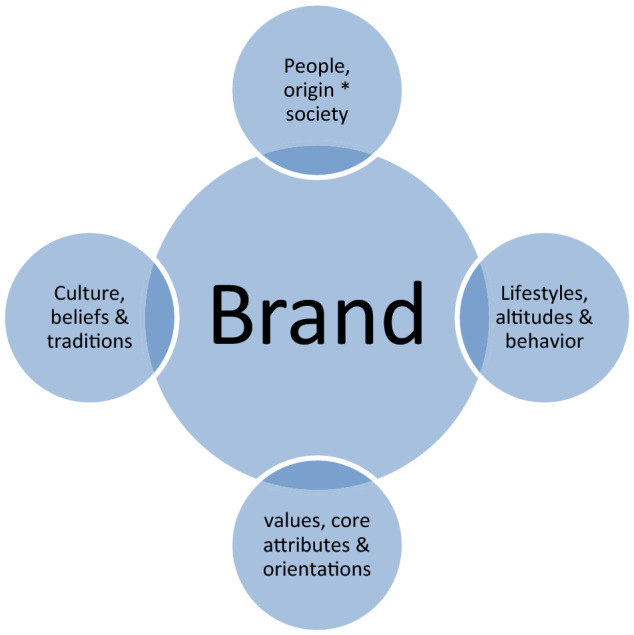
Brand as a tie, connection, and association at the center between the people, lifestyles, and their cultures; source: developed and drawn from author’s observation based on this study (2019–2021)

In light of this presentation and present study and observation embedding “investment and climate change risks” into the proposed business and sustainable marketing model (author’s draft in 2019 and present study) is crucial as presented in Fig. 5 subsequently below

As enumerated by Loosemore and Lim (2018), there were four dimensions of CSR that can be explained with the help of a pyramid: economic responsibility, legal responsibility, ethical responsibility, and philanthropic responsibility. In this light as unveiled, CSR has a deep and positive impact on corporate image and reputation (Lu et al. 2019). Studies further found that consumers’ participating in charitable donations through the CSR activities organized by enterprises could help to improve the image of product brands (Luo and Lv 2019), thus justifying hypothesis formulations 1 and 2 as presented above, which can translate to optimized use of resources for organizations that adopt CSR in their business models, and imbibed as a strategy.

Charitable donations had a greater effect on consumers’ evaluation of an enterprise than business sponsorship (Liu 2014). They had an effect on consumers’ continuous purchasing willingness (Feng et al. 2019), which could enhance the perception of consumers' corporate reputation (Szőcs et al. 2016). Similarly, the volunteer activities of an enterprise also had a positive impact on the corporate image (Plewa et al. 2015). Enterprises can effectively combine society, the economy, and employees by organizing and implementing volunteer activities (Zhou and Lu 2011). Therefore, enterprises should actively organize CSR activities to make consumers perceive CSR (Dawood 2019) and thus affecting the corporate brand image. Based on the above analysis, the hypotheses above are justified as proposed and presented.

### Nature of the study and research design

As briefly stated earlier in the introduction to this research, a qualitative-based succinct description with novel model presentation and quantitative approach would be sought by way of acquisition and analysis of the primary data source, thus, depicting this research approach as a “mixed – method”.

The research would be conducted based on quantitative methods, while drawing extensively from grounded theory and literature presentation, making vivid qualitative analysis and quantitative treatments from inferential statistical methods applying data acquired from questionnaire administration and survey.

A quantitative approach is an objective, fact-finding process that is based on clear evidence and records. As explained in Dawson (2007), quantitative research is known and characterized by the generation of statistics through large-scale surveys, and on the other hand, a qualitative approach is a subjective process that aims to explore attitudes, demeanor, experiences, and opinions of the participants, displaying reflexivity from the interviewer, participants and respondents or reflections.

Therefore, based on the descriptions and explanation and reinforcement, a mixed approach was selected and adopted following the explicit literature, concepts, and grounded theories as the research approach of this paper, which gave a detailed understanding and clarification of the research problem. In agreement with Amaratunga et al. (2002), adopting a mixed research approach counteracted the weaknesses or fractures and shortcomings of quantitative and qualitative approaches, and as mentioned earlier for reinforcement. As defined by Sandelowski (2000, p. 254), mixed-method research is a “dynamic option for expanding the scope and improving the analytic power of studies”.

A combined, or mixed method allows better insights into the research and can enable seeing a clearer picture of where qualitative and quantitative methods contradict or agree (Shorten and Smith 2017).

The primary data source requisite for this study would be acquired via questionnaires administration, field or experimental survey, and simple random sampling distributed; by random sampling, each sample or participant has an equal likelihood or chances of being picked or selected and cutting across the selected the various industry vertices or verticals randomly drawn across consumers and participants in the presented industry vertices. This would involve a slightly rigorous quantitative treatment and acquisition of the primary data salient for the conduct and accomplishment of this research task.

Subsequently, having acquired or after acquiring all the important primary data, the next step of this research investigation is the quantitative treatment of the primary data acquired from the survey and experimental field following a qualitative explicit literature description, model proposition, and hypothesis formulation built around the research questions.

The quantitative treatment of the data is statistically based and done by verifying the validity or non-applicability of major hypothesis assumed or set in the course of this research investigation.

Finally, the quantitative treatment as demonstrated by the SEM: “structural equation model” analysis establishes a correlational relationship between the variables, or a pair comprising a dependent and independent variable, such as a brand with CSR, and investment risks, and might be described as partly causal, as well as correlation as demonstrated and carried out in the ANOVA, and composites or embedded variables and terms.

**Data sources**: The data was acquired as mentioned above based on random sampling or probability sampling. Samples were based on questionnaire administration taken in Rome and neighborhood capturing 125 responses from respondents. An additional 50 questionnaires were administered equally across the public and organization domains to capture perceptions on brand and CSR as key components of organizational culture and structure as analyzed from the ANOVA subsequently. The total sampled population totaled 175.

By the rule of ‘thumb, a minimum of 30 sample size is sufficient for a population sampling; in representativeness of the data and population however, a total of 225 questionnaires in all were administered.

**The representativeness of the data, sample sizes or sample distributions and sampling**:

The representativeness of the data or sample size and selected population follows from the expression;

$$n=\frac{\frac{{z}^{2}\times p\left(1-p\right)\times N}{{e}^{2}}}{N-1+\left(\frac{{z}^{2}\times p\left(1-p\right)}{{e}^{2}}\right)}.$$

Prior to the sampling; envisaging a population size of 400–500; the representativeness of the population size is a sample size of 162, 170, and 176 based on populations of 400, 450, and 500 adopting the expression above.

As shown in Table 4), the Cronbach alphas lie between 0.78947 and 0.92308, which far exceeds the threshold, and is relatively high, hence justifying the reliability of the variables and parameters or measures or assessment.

## Findings and results

The table of results is prepared based on data acquired from a poll, interview, opinion capture, and extensive fieldwork-based survey done in **Rome** between May 2018 and December 2019 extended all through the pandemic phase of 2020 till mid-August (Table 1).

**Table 1:** Field survey

Observations	Frequency
Climate change (aware):	121
None	4
Conscious of a brand:	120
Not known or varies	5
Recycling awareness	121
Racolta differenziata	78
CSR as a brand (support)	121
Not sure or varies:	4
CSR and environmental sustainability (partial connection)	
Agree	78
Disagree	32
Not sure	15

Statistics/summary: number of administered data sheets: 134

Number of observations: 125

Feedback not gotten yet: 9

Table 2 is an extension of the poll to investigate CSR and brand relationship capturing 50 questionnaires comparing corporations and public domain perceptions while Table 3 is the extension of the poll furthermore in which additional 50 data sheets and questionnaires were captured prior to March 2020 and during the pandemic peaks till ‘mid-August of 2020 to capture more fields in addition to the previously administered questionnaires poll done up to December 2019 as earlier mentioned above, bringing the additional polls to a total of 100 in the pandemic, through the peaks and post-pandemic periods till 2021 (Table 4).

**Table 2:** Field Survey

Observations	Frequency (corporations)	Frequency (public)
Conscious of a brand	25	22
Not known or varies	–	3
CSR as a brand (support)	24	23
Not sure or varies	1	

Statistics/summary: Number of administered data sheets: 50

Corporations: 25

Public: 25

Number of observations: 50

**Table 3:** Field survey

Observations	Frequency
Previous participation in climate redress	–
None	50
Investment risks as mitigating tool	47
Not known or varies	3
Optimization from investment risks	38
Depends	12
Investment risks (compensation)	45
Others	4

Statistics/summary: number of administered data sheets: 50

Number of observations: 50

Sources of Tables: Present study draft and author’s draft

### Reliability test and validity

The reliability test and validity assessment are done from the ‘Cronbach; alpha as presented in the following table.

As shown in Table 4, the Cronbach alphas lie between 0.78947 and 0.92308, which far exceeds the threshold, and is relatively high, hence justifying the reliability of the variables and parameters or measures or assessment.

**Table 4:** Cronbach alpha of the variables

	Cronbach alpha
Brand awareness	0.78947
Investment risks and culture	0.83768
Climate change and investment	0.84906
Investment risks and brand	0.92308

Source: Present study draft and author’s draft

### Emerging model(s): presenting CSR conceived as a brand CSR as a brand, implications, and bearing

CSR as a brand is a crucial and central playing position that connects the consumers and manufacturers, company or marketing teams, and society and is potentially useful in building a strong relationship between the entire parties and as a potential check against a number of unwholesome marketing practices and abating climatic changes and environmental impacts of businesses on the environment and society.

Pointing out these crucial factors, Abbes et al. (2020) mentioned that enterprises should be aware of the importance of the implementation of CSR and as means of improving their brand satisfaction; and brand identity (Luo and Jiang 2019) while also recognizing consumer brand participation (Kaur et al. 2020), thus actively influencing brand loyalty.

This is a good bearing and direction to tread and pathway to pursue in my opinion: a well-branded organization within a CSR perspective and strategizing would capture profits in returns from loyalty brand building and well position in its brands.

This fact and bearing further points to the ‘justification of the new model I have carved and presented in Fig. 4 above.

CSR as vividly highlighted by its component mix(s) and combinations seen in the figure from the perspective of a brand connects the company, society, and consumers and portrays a potential facilitator and strategic positioning tool for the brand. This is because a well-positioned brand within a CSR context can draw and attract increased brand attractiveness, affection, and loyalty as consumers are liable and most likely want to associate with such brands that show and portrays socially responsible traits as seen as brands who care for their well-being, community and pursue overall good interests of the society at large and as such would want to maximize their trusts in such brands and affections.

#### Chart/schematic(s)

Fundamentally, the principle of societal marketing asks the company to consider the “consumers” wants or desires and long-term interests, the company requirements, and society’s long-term interests (Armstrong and Kotler 2008).

As seen CSR merged conceived as a brand or identity is like a connecting duct, wire or bridge central and key position that ties and act as a nexus common to the parties consisting of the company, manufacturers or marketers, society, and the consumers.

In this light and revelation; the following model emanated from this study as presented:

As shown in Fig. 4: CSR as central and key to the company, consumers, and society, and following the hypothesis framework and extrapolations from the working template, CSR is crucial and key to the company, consumers, and society, in actual fact, CSR can significantly shape the brand, from brand reputation to brand equity, consumers can show more inclinations and affections for their preferred brands, this will significantly and positively impact on the company who can gain from returns and society at large and environment. If organizations take CSR as a priority and imbibe it as a business model, the environment can be saved from environment-related courses and will have an impact on climate change and mitigation.

Presenting CSR as a brand and thus conceived justifies the relationship and community approaches as some of the key concepts to branding and brand management (Aniket 2014). Saying further CSR, brand, and well positioning can be the basis for value creation and creating consumers’ satisfaction, establishing a closer brand relationship with consumers and society by caring for their well beings.

Finally, the emerging model as illustrated in the chart can foster a long-term relationship between the companies and consumers who promise strong brand loyalty to companies that turn out socially responsible products within the context of value-based marketing and orientation.

Companies can adopt CSR as a brand and merge appropriately with their business strategies within a value-based and socially responsible or societal marketing orientation through the right strategies and devices embedding ‘investment risks forming the major theme of this research as mitigation against climate changes and environmental impacts of negligent practices from industrialization as supported by this model to the long term goals and capture profitability in returns.

### Test 1 or verifying assumption

Based on previous data; Is there a connection between the brand image identity and the corporate social responsibility roles of an organization?

It is expected and by the underlying assumption prior to the survey and previous experiences a minimum of 75% of respondents agree with this claim of brand connection, image identity, and CSR roles played by reputable organizations, this is set to the null hypothesis while the contrary is the alternative hypothesis that this claim is not true or should be rejected.

**Null hypothesis**:

$${H}_{0}:p=0.75.$$

The null hypothesis is the working assumption that a minimum 75% of respondents agree or conscious opinion of a strong relationship between brand image and the roles an organization plays in its corporate social responsibility, overwhelmingly found in this investigation all respondents interviewed agreed with this position presently.

**Alternative hypothesis**:

$${H}_{1}:p\ne 0.75.$$

At the; 10% level of significance;

$$CV: 1383,$$

where;

$$\widehat{x}=1.0.$$

Following the calculation, formula or expression given above for *z*-stat;

$${z}_{\mathrm{cal}}=\frac{1.0-0.75}{\sqrt{\frac{\left(0.75\right)\left(0.25\right)}{125}}}=\frac{0.25\times 25}{\sqrt{0.1875}}\approx 14.4342,$$

$${z}_{\mathrm{cal}}>{z}_{\mathrm{tab}}\left(\mathrm{CV}\right).$$

Since *z-*stat or ${z}_{\mathrm{cal}}$, from the formula is greater than CV or tabulated value, we accept the null hypothesis that a minimum of 75% of respondents agreed with the existence of a strong connection between the brand image and CSR: corporate social responsibility roles of an organization or company.

Inferring from the test 1 hypothesis verification and based on this evidence affirmed statistically there is a relationship between CSR and the brand image of an organization. In connection and bearing with test hypothesis 2 CSR would enhance the brand image of an organization, image, and quality or brand equity and supported by the emerging model presented and illustrated in Fig. 1 above and subsequently expanded upon in Fig. 2 showing explicitly the brand connections among highlighted mix(s) and furthermore in Fig. 3 highlighting its implications incorporating investment risks by adopting a value-based marketing and socially responsible or societal marketing orientations.

**CSR and brand relationship: organizations vs. public domain perceptions!**

Based on subsequent data, pooling the variances for the organizations and public domains and setting two hypotheses made up of the null and alternative;

**Null hypothesis**

$${H}_{0}: {\mu }_{1}={\mu }_{2}.$$

The null hypothesis as earlier mentioned and elaborated further is the working assumption that the means are equally based on the two-sample independent means *t*-test with unknown population standard deviations and equal variances assumption following the ‘Likert ratings based on the recent interview and poll conducted during the investigation and supporting or close to the assumptions based on the responses.

**Alternative hypothesis**

$${H}_{1}:{\mu }_{1}\ne {\mu }_{2}$$

At the; 5% level of significance; applying statistical tools and testing based on software application the following table is obtained as presented with detailed results (Tables 5, 6).

**Table 5:** Table showing the statistics

*N*	*df*	Mean 1	Mean 2	SD 1	SD 2
50	48	4.9804	4.8945	0.8821	1.9057

Significant finding; *p* > 0.05

**Table 6:** Table showing the statistics

*N*	*df*	Mean 1	Mean 2	S.D 1	S.D 2	Std.Error (*σ*_error_)	*t*-critical	*p* value
50	48	4.9804	4.8945	0.8821	1.9057	0.2788	2.011	0.8388

Conclusion/comments: The intermediate calculated value is 0.205

The *p*-value is *p* = 0.8388, and since *p*-value is greater than or equal 0.05, the null hypothesis is not rejected

The 95% confidence interval for the population mean is;

$$- 0.{759} < \mu_{{1}} - \mu_{{2}} < 0.{93}.$$

In line with the research question and the ‘hypothesis drawn in line with a brand relationship as a key component to be embedded in the brand strategy and CSR and expressing the relationship between brand and CSR; a relatively high likelihood of most members of the public domain seeing this perspective in strong co-relation, bearing an agreement with the top management team position and employee below the management cadre as subsequently pooled.

The relatively high mean justified this fact or bearing.

**N.B:**

The additional poll and respondents were drawn in the earlier part of the pandemic as mentioned earlier to express further the relationship between CSR and brand pertinently comparing the perspectives of organization and the public domain.

**Investment risks in climate change mitigation**

Investment risks appear to be an extremely key and significant consideration to organizations in the present state and emerging realities associated with climate changes and resulting consequences manifesting in rising incidences of litigation and legal suits against corporations, governments, and organizations seeking redress and compensations for harms or damages induced through the company, organization, and government activities on the community concerned as subsequently enumerated and succinctly discussed with some notable references.

Most times and often companies, organizations, and governments affected and in the position of facing and answering one legal suit or litigation often and usually seek to look for bailouts and ways to overturn cases lobbying in their favor of successive judgments and file suits against such and do eventually spend some considerable anoint of funds and resources that might be seen as wasteful.

If corporations are more socially responsible in their approach and by pragmatism adopt and imbibe into their culture investment risks these resources rather than being plunged and wasted pursuing legal suits filed or jury cases against such can be better channeled into corporate donors and socially responsible packages for the communities where they operate and dwell in.

**Perspectives from public domain and organizations!**

#### Test 1 underlying basis or verifying assumption

##### Assumptions

There are high probable chances and tendencies for a larger number of respondents to favor or support investment risks for its benefits in abating climate changes but there might be some fewer contrasts!

I’m pooling the two response fields obtained and captured in this investigation from both the consumers and investors; organizations and public sides by a two-sample pool of means as done below.

*P*_1_ is the proportion from organizations and *P*_2_ is the proportion from the public domains.

**Null hypothesis**

$${H}_{0}:{p}_{1}={p}_{2}.$$

The null hypothesis is the working assumption that a large relatively high proportion of respondents from corporate bodies, organizations, employees with top management and public domain agree that investment risks can significantly contribute to climate change mitigation and prompt achieving it.

**Alternative hypothesis**

$${H}_{1}:{p}_{1}\ne {p}_{2}.$$

At the; 10% level of significance;

$\mathrm{CV}:-1383,$where and setting;

$${p}_{1}=0.93, {p}_{2}=0.96.$$

Following the calculation, formula or expression given above for z-stat;

$${z}_{\mathrm{cal}}=\frac{0.93-0.96}{\sqrt{\frac{\left(0.93\times 0.07+0.96\times 0.04\right)}{25}}}=\frac{-0.03\times 5}{\sqrt{0.1035}}\approx -0.4662,$$

*p*-value (one – tailed): 0.32088; *p* value (two – tailed): 0.64175.

$$\left|{z}_{\mathrm{cal}}\right|<\left|{z}_{\mathrm{tab}}\left(CV\right)\right|.$$

Since *z*-stat or ${z}_{\mathrm{cal}}$ from the formula is greater than CV or tabulated value for the left tail test, we cannot reject but accept the null in line with our expectation and initial assumption and hypothesis that a majority of respondents agreed on the existence of a strong connection and correlation between the investors, corporate organizations, employees public domain on the perception of investment risks and potential or significance in climate change mitigation, combating related challenges brought by or induced.

These results and findings align with one of the key elements of CSR, which is the environment of the firm (Loosemore and Lim 2018; Irshad et al. 2017), which includes the ambiance, aesthetics, buildings, and decor. As its implication as unveiled; Wu and Wang (2014) suggested that firms with good CSR usually had higher returns and profits. Kotler and Lee (2005) suggested CSR as a commitment to society.

#### Further verification and testing

As mentioned above in line with the assumption of the null hypotheses and testing on another side mostly over 75% of respondents however to a proportionally and relatively high degree or extent agree to the opinion of making their investments risks key component of CSR, brand and corporate structure.

Past experiences, conditions, and risk level perceptions or uncertainties, accordingly may overwhelmingly influence perceptions, especially with the pandemic regime that overlaps this research's second stage of the data acquisition phase.

This hypothesis is subject to further test verification, based on *t*-test and adopting the scaling or Likert rating scales and applying scale (1–5): 1—less agree or not sure, 2—mildly agree, 3—quite agree, 4—strongly agree, and 5—very strongly.

While findings and investigation from the polls showed 75% agree with this claim and hypothesis, the investors and management teams that CSR can play a key and dominant role in climate changes mitigation and that incorporating investments and climate change risks organizations can achieve optimal resource utilization in agreement with the general public and employee below the management cadre up to 92–94% to another relatively and proportionally overwhelmingly high 96.0% bracket range ‘though exceeding the responses from organizations as further elaborated below from Table 9) results that investment risks should be embedded into the brand as a culture perceived based on responses garnered and acquired from another section of the structured questionnaire and interview poll administered in the course of this investigation.

### Investment risks in climate change mitigation

Investment risks appear crucial as presented in this study and established further as a key element to be embedded in the culture and brand following the hypothesis presentation and the research questions:

The brand is significant and has been investigated extensively from literature and on how it connects with CSR (Tables 7 and 8).

**Table 7:** Table showing the statistics

*N*_1_	*N*_2_	*df*	Mean 1	Mean 2	SD 1	SD 2
25	25	23	3.884	4.985	0.7562	0.9094

Significant finding; *p* > 0.05

**Table 8:** Table showing the statistics

*N*_1_	*N*_2_	*df*	Mean 1	Mean 2	SD 1	SD 2
25	25	23	3.884	4.985	0.7562	0.9084

Significant finding; *p* > 0.05

**Null hypothesis**

$${H}_{0}: {\mu }_{1}={\mu }_{2}.$$

The null hypothesis as earlier mentioned and elaborated further is the working assumption that the means are equally based on the two-sample independent means *t*-test with unknown population standard deviations and equal variances assumption following the Likert ratings based on the recent interview and poll conducted during the investigation and supporting or close to the assumptions based on the responses that test a minimum 75% respondents from the organizations and public domains agree on investment risks as a proactive and potential check or mitigation and abatement against climate changes and overwhelmingly relatively and proportionally high up to 92–94.0% respondents agree with this position and seemingly divergent as seen from the table of statistics presented in 1a) the public domains show higher means accounting more for the overwhelming 96.0% pooled.

**Alternative hypothesis**

$${H}_{1}:{\mu }_{1}\ne {\mu }_{2}$$

At the; 5% level of significance; Applying statistical tools and testing based on software application the following table is obtained as presented with detailed results (Table 9).

**Table 9:** Table showing the statistics

*N*	*df*	Mean 1	Mean 2	S.D 1	S.D 2	‘Std.Error (*σ*_error_)	*t*-cal	*p*-value
50	48	3.881	4.945	0.7562	0.9084	0.16604	4.67	0.000

Conclusion/comments: The critical value for a two – tailed tail test here is *t*_*c*_ = − 2.011

The *p*-value is *p* = 0.000, and since *p*-value is not greater than or equal to 0.05, the null hypothesis is not accepted but rejected

This overturns the initial assumption of the null hypothesis even though both respondents drawn from both the organizations and public domains agree to varying extents on investment risks adoption as a tool and means or check to abate and mitigate climate changes. This implies that the opinions and positions of public domains and organizations might be different, even when both support investment risks.

The fact that the public seems to be relatively high in favor of this position might be the strong basis and evidence asserting the rising incidences in litigation cases and jury suits emerging quotidian and frequently on the increase in seeking redress against harmful practices against the environment by firms, organizations and government as reflected and evidently seen.

The 95% confidence interval is;

$$- {1}.{379} < \mu_{{1}} {-}\mu_{{2}} \, < - 0.{629}.$$

**SEM: investment risks and climate change mitigation**

A structural equation model (SEM) is presented and implemented to establish and affirm the proposed relationships and also support the models and novel frames captured as subsequently elaborated.

Dependent variable – investment risks.

Independent variable – climate change mitigation (Table 10).

**Table 10:** Regression table on climate change and investment risks

Parameters	*β*	*R*^2^	*α*
Climate change – Investment risks	0.5596	0.8286	2.8243

$$CC=\alpha +\beta .IR.$$

The above equation expresses the relationship between climate change and investment risks from regression – fit; a high R-squared value as shown in the table of 0.8286 implies and justifies the fact that investment risks can be a strong, veritable and potential tool to curb and lessen climate changes as inferred from this result and the data captured on the opinion polls from the respondents.

If corporations, industries, and organizations at large take strong cognizance of the environmental impacts of the activities, climate, and society at large and imbibe an altitude of putting investment risks into their strategies in the sense of social responsibility by promoting and supporting friendly practices they would do all possible best and efforts to avert and reduce negative aspects of their productions that can adversely impact the environment.

Furthermore, such corporations and organizations in an environmental and ethical or moral sense would take utmost priority in protecting the social-well-being of the communities they operate and make investment funds and contributions towards welfare packages and incentives for communities in providing social amenities and infrastructures to better and enhance their lifestyles.

Like a hedge against risk, such incentives and funds would go into savings and positive use to life impactful projects in the communities and societies rather than going into litigations and jury suits in seeking redress against reprimands and actions or legal suits brought against corporations by communities such as the Urgenda as subsequently mentioned and elaborated.

Finally, the ‘SEM is presented.

**SEM**: **investment risks and climate change mitigation**

The existence of a statistical significance indicates that investment risk can be a viable tool, strategy, and instrument for climate change mitigation within the core claims of this study.

#### CSR, brand relationship and investment risks

There is strong support as revealed and evident from the hypothesis testing between management teams, employees below lower cadre, and the public on investment risks as a culture to be embedded into the brand strategy:

Investment risks can potentially and greatly safe-guide towards resource loss and wasteful spending unnecessarily on pursuing litigation charges brought against corporations rather than plowing those resources into improving the well-being and social life of the people, consumers, and society.

Discussing further companies and corporations that expresses good and strong brand relationship is highly probable to adopt investment risks as a potential tool and mechanism for boosting relational marketing, responsible business attitude socially, and giving back to society.

#### Investment risks and brand relationship

**Proposition/assumption**

There is supposed to be a strong correlation between investment risks and the brand relationship or image.

Consumers, the public and society would most probably associate with corporations that incorporate investment risks into their brands; this would enable building a strong brand with a reputable image and establishing a good and friendly brand relationship.

This led to the following regression fit between the investment risks and brand relationship:

$$BR=\alpha +\beta .IR$$

**SEM**: Investment risks and culture, CSR and brand reputation:

Pertinently, the statistical significance shown based on the *p* value (< 0.0001) from Table 13 is an indication that there is a strong connection between investment risks and culture, CSR and brand reputation following the high R Sq. – value observed and seen from Table 12.

**Investment risks as culture**

The 95% confidence interval for the population mean is;

$$- 0.{\text{393}} < \mu _{{\text{1}}} - \mu _{{\text{2}}} \, < \,0.{\text{311}}.$$

In line with the research question and the hypothesis drawn in line with investment risks as a key component to being embedded in the brand strategy and CSR, a relatively high likelihood of most members of the public domain seeing this perspective in strong co-relation, bearing an agreement with the top management team position and employee below the management cadre.

The relatively high mean of being fervent and supportive of investment risks as a culture by the public and organization and more pertinently public showing a slightly higher mean might be strongly demonstrative and supportive of the research question posed to the public that optimization of resource utilization and resources saved or optimized by organizations would go into corporate philanthropy or charity especially in my observation the fact that this poll was done during the COVID-19 and pandemic peak period when society and the public most likely want something from corporations by corporate philanthropic spending and palliatives.

**ANOVA test**:

In a further presentation and analysis of the ANOVA one-way test was done to compare and do a variance analysis of two grouped data captured from the management team and public domain:

Perception of the management team, employee, and public domains on investment risks, climate change, brand culture, and relationship:

A composite poll was conducted as included in the questionnaire to capture the general and overall perception and consciousness level of the corporate organization, firms, employees, and public domain on the relationship between brands and investment risks. Climate change and brand culture’s relevance are tied with investments as a culture to be drafted into the brands and CSR.

The outcome of this composite poll and data capture is presented based on the data summary and ANOVA statistics explicitly summarized below:

**Observations and ratings**: **based on Likert ratings (1 – 5)**

**Calculations**: **ANOVA estimates**

**DATA summary:**

**ANOVA summary**:

**Comment**(s)

As seen above the *p* value (> 0.05), it implies the assumption of the composite relationship existing and connecting the brands and investment risks; investment risks as a part of brand culture and the overall perception shown and demonstrated by the corporate organization, top management team or echelons, employees and public domain are perceived to be overall high.

As opined and recognized by Loosemore and Lim (2018), there were four dimensions of CSR that can be explained with the help of a pyramid: economic responsibility, legal responsibility, ethical responsibility, and philanthropic responsibility.

In view of the literature and consistent with the present study and findings, deductively, CSR has a deep and positive impact on corporate image and reputation (Lu et al. 2019). Studies further found that consumers’ participation in charitable donations through the CSR activities organized by enterprises could help to improve the image of product brands (Luo and Lv 2019). This is supported by the SEM results, and regression model–fit models on relatively high *R*–Sq. values, and statistical significances from Tables 11, 12, 13, 14 and 15, then Tables 16, 17 and 18 from the ‘ANOVA, justifying a high and strong correlation between CSR and brand with the variables examined, and pointing strongly to significances and the potential impacts of CSR as a potential tool, instrument and strategy in mitigating climate change risks, optimization and addressing key issues related to the environment, society and the planet entirely in driving towards a sustainable future, greener and safer planet.

**Table 11:** Brand reputation from CSR - investment risks, brand and culture

Parameters	*β*	*R*	*t*	*p* value	SER_β_
Brand reputation (CSR)—investment risks and culture	0.5596	0.91027	1.8206	< 0.00001	0.3073

Comment: The result is significant at *p* < 0.05

**Table 12:** Brand relationship and investment risks from regression analysis

Parameters:	*β*	*R*^2^	*α*
Brand relationship – investment risks	0.3522	0.9516	3.3862

**Table 13:** Brand reputation and investment risks from ‘SEM: Structural equation modeling

Parameters	*β*	*R*	*t*	*p* value	SER_β_
Brand reputation (CSR) – Investment risks and culture	0.9977	0.9753	2.2538	< 0.00001	0.4427

Comment: The result is significant at *p* < 0.05

**Table 14:** Table showing the statistics

*N*	*df*	Mean 1	Mean 2	SD 1	SD 2
50	48	4.804	4.945	0.6521	0.9157

Significant finding; *p* > 0.05

**Table 15:** Table showing the statistics

*N*	*df*	Mean 1	Mean 2	S.D 1	S.D 2	Std.Error (*σ*_error_)	*t*-critical	*p*-value
50	48	4.804	4.945	0.6521	0.9157	0.06521	2.011	0.5335

Conclusion/comments:

The intermediate calculated value is 0.627

The *p* value is *p* = 0.5335, and since *p*-value is greater than or equal to 0.05, the null hypothesis is not rejected

**Table 16:** Ratings & observations based on ‘LIKERT Scale

Group 1 Observation: (ratings)	5	4	3	4	5
Group 2 Observation: (ratings)	4	5	5	4	4

N.B: Group 1: Organizations, corporations and firms

Group 2: Public domains and consumers

**Table 17:** Descriptive statistics from the group comparisons

	*N*	Mean	Std. dev	Std. err
Group 1	5	4.2	0.8387	0.3742
Group 2	5	4.4	0.5477	0.2449

**Table 18:** ‘ANOVA: Summarized statistics from the two groups comparison

Source	*df*	SS	MS	*F*	*p*
Between Groups	1	0.1	0.1	0.2	0.6666
Within Groups	8	4.0002	0.5		
Total	9	4.1002			

*p* value: 0.66659

*F*: 0.2449

Charitable donations had a greater effect on consumers’ evaluation of an enterprise than business sponsorship (Liu 2014). They had an effect on consumers’ continuous purchasing willingness (Feng et al. 2019), which could enhance the perception of consumers' corporate reputation (Szőcs et al. 2016). Similarly, the volunteer activities of an enterprise also had a positive impact on the corporate image (Plewa et al. 2015). Enterprises can effectively combine society, the economy, and employees by organizing and implementing volunteer activities (Zhou and Lu 2011). Therefore, enterprises should actively organize CSR activities to make consumers perceive CSR (Dawood 2019) and thus affecting the corporate brand image. Based on the above analysis, results and findings as fully presented and in line with literature revelations the hypotheses, within underlying assumptions are as proposed:

**Co-relation matrix**

A correlation matrix from the variables (Tables 19, 20);

**Table 19:** Correlation matrix of variables

	CSR	Brand reputation	Brand image
CSR	1.0000		
Brand reputation	0.9476	1.0000	
Brand image	0.9378	0.9365	1.0000

**Table 20:** Correlation matrix of variables

	CSR	Investment risks and brand	Climate change and investment	Investment risks and brand
CSR and brand	1.0000			
Investment risks and culture	0.9487	1.0000		
Climate change and investment	0.7746	0.8165	1.0000	
Investment risks and brand	1.0000	0.9487	0.7746	1.0000

As shown in the table above from Table 21; to test the normality and assumptions of normal distribution set in the ‘hypothesis testing and verification process and affirmation of the assumptions; the values of the kurtosis and skewness all fall within the range of − 1.96 to + 1.96, thus justifying the assumptions of normal distributions.

**Table 21:** Parameters and verification of normal assumptions to hypothesis verification

Parameter or variable	Mean	Standard deviation	Skewness	Kurtosis
Brand awareness	3.79	0.3742	− 1.426	1.939
Brand relationship – brand reputation	3.9	0.1414	− 1.364	1.321
Investment risks (climate change mitigation)	3.5	0.3742	5.961*e *− 17	− 1.2
Investment risks (culture)	3.7	0.3162	− 1.186	1.05
CSR (environment)	3.738	0.2669	− 1.205	1.492

The data sets on average and in most instances are less skewed and less tailed to the left or right showing high peaked behavior and fit for normal distribution and suitability of the ‘hypothesis testing and verifications.

**CVM: common variance method and common methods biasing**

Common method biasing as it indicates points to biases in the measurements and relationship between the variables.

The discriminant validity expresses if there is discrimination between the variables or not, and to what extent.

The HTMT: hetero-trait mono-trait correlation determines the variances and variation between the measured observations and actual responses from the respondents or subjects. The threshold assigned is a value of 0.85. There is a discriminant between the variables if the value is below the threshold, and non if the value exceeds or is relatively far above or exceeding 1.0.

The HTMT 2 expresses further validity. The latent variables are hidden measures or parameters associated with the variables and measures. A latent variable associated with CSR and Brand might be logo.

In this case, the latent variables associated with CSR, brand reputation, and image are considered as; “Investments risks and culture, climate change and investment risks, investment risks and brand, and brand awareness”.

**Discriminant validity**: **measures of discriminant validity based on HTMT**

The ‘HTMT & HTMT 2 Tables as shown from 23 and 24 below are produced from the latent variables considered to examine the validity, and ascertain or establish the presence or absence of a discriminant between the variables, expressing validity or not.

LV1 & LV 2 are the designated latent variables consisting of 2 items each listed in Table 22 as; brand awareness, investment risks & culture, then ‘climate change & investment, and investment risks & brand.

**Table 22:** Cronbach alpha

	Cronbach alpha
LV 1	0.97367476
LV 2	0.87298546

**Table 23:** HTMT

HTMT		
LV 1		
LV 2	1.03232284	
	LV 1	LV 2

**Table 24:** HTMT 2

HTMT 2		
LV 1		
LV 2	1.0268585	
	LV 1	LV 2

**N.B**:

HTMT: Hero-trait mono trait correlation. The HTMT2 is the improved calculation and value, but usually smaller than HTMT.

The results indicate that; there is no discriminant between the variables and indicates a significant level of validity as the HTMT and HTMT2 values of 1.03232284 and 1.0268585 exceed the threshold value of 0.85 in the two instances or cases.

Also, based on the Cronbach alpha of 0.97367476 and 0.87298546 from the latent or hidden variables, there is a strong correlation, as this exceeds a minimum or moderate range, the values are relatively high indicating and expressing strong correlation and association between the variables, and items, implying reliability of the associations examined and the variables.

## Discussion

Social equality is a highly demanded social change. Inequality has been linked with capitalism and a massive craving for wealth or accumulation.

Capitalism in the twenty-first century, wealth accumulation and holdings still put resources and massive wealth control in custodians and in the hands of a few as was prominent in eighteenth-century American society.

The call for social equality and addressing inequality is in high demand and virtually urgent.

CSR interestingly has further assumed multidimensionality as unveiled and evident in literature (Crane and Kazmi 2010; Kristic 2017; Kristic and Piper 2020; Martin-Ortega and Wallace 2013) with attention and focus even put on pushing and demanding human rights, gender issues redress, and labor, child abuse and rights, etc.

Slobodan Marić et al. (2021) presented a study on relations between corporate social responsibility (CSR), employee commitment, and firm performance in Serbia.

The theoretical part of the article analyzes the relationship between CSR and firm performance, as well as CSR and employee commitment, based on available worldwide research results. The empirical part of the article presents the research results for large companies in Serbia, regarding the relations between CSR and firm performance, CSR and employee commitment, and the mediating role of employee commitment on the relationship between CSR and firm performance.

The authors determined that there was no direct effect of CSR on firm performance, but positive, statistically significant effects on employee commitment. Although the direct effect is missing, employee commitment has a positive mediation effect on the CSR-firm performance link.

### CSR model(s)

**Fig. 6:**
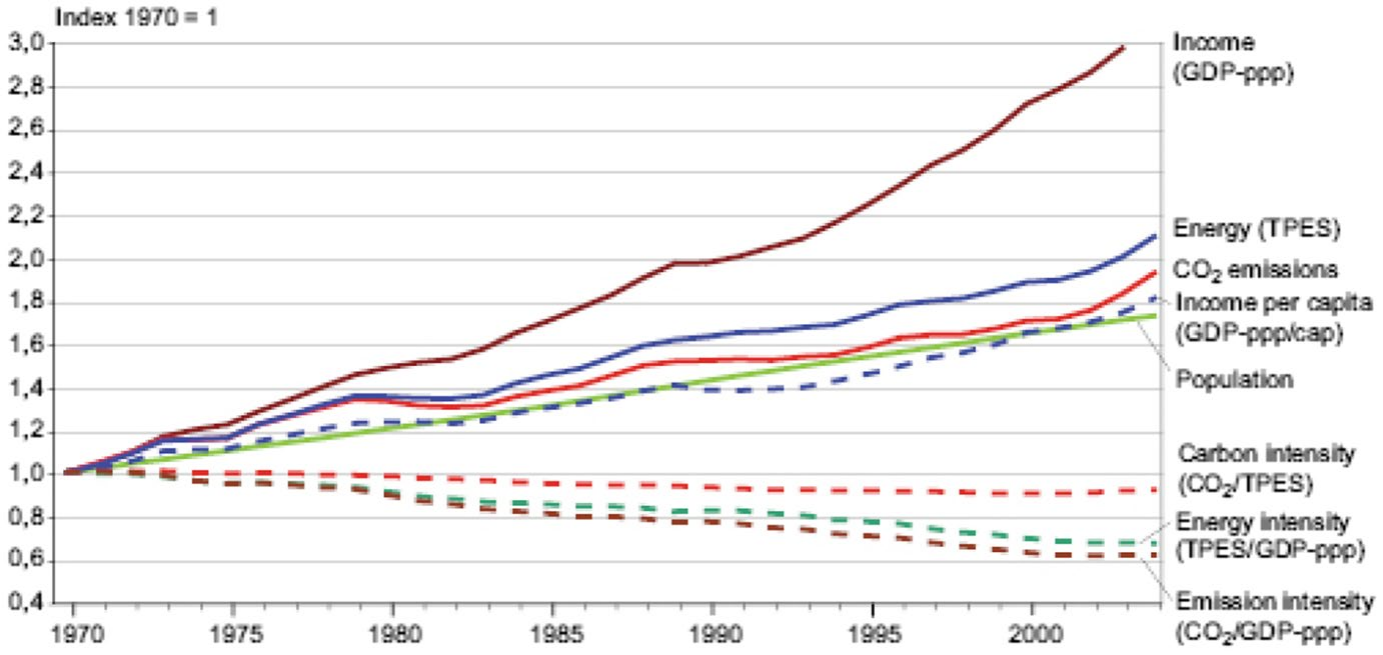
Global greenhouse emissions

Drawn and based on observation and the present draft, key models have been presented in Figs. 3, 4, and 6 constitute the basis and framework on which this study is built. Another model as carved in its novelty is presented in Fig. 5.

The carved models have been justified by the findings and results presented all through this discussion and significantly contribute to the literature that CSR has tremendous roles to play; conceiving CSR as a brand, a company can enhance its brand, as consumers develop a tie as expressed in Fig. 4, doing so companies can be more careful not to violate legal guidelines and involve in virulent practices against the environment.

CSR as a business model helps companies to self-regulate activities that impact stakeholders, including the general public (Ferrell and Hartline 2011). This is strongly justified and supported by ANOVA results in Table 16—con the perceptions of public domains and organizations as both parties crave and show a penchant for CSR, more importantly as a potential strategic tool and instrument in checking and mitigating climate changes and negligent practices that might adversely or severely impact on the environment.

CSR goes simultaneously, hand in hand, and is strongly associated with a smart brand strategy as implied and evident.

As shown in Fig. 6: a brand is a tie, connection, and association at the center between the people, lifestyles, and cultures.

Also from the presentation of the hypothesis; it is been clearly outlined that CSR can significantly enhance the brand, and drive a key motivation and enthusiasm in the consumers, being consistent with the literature as enumerated (Lu et al. 2019; Mahmood and Bashir 2020) among others, while playing significant roles as well (Kotler and Lee 2005; Kotler et al. 2018).

The entire brand attributes highlighted in the novel model presentation from Fig. 6 and potential benefits to corporations, consumers, and society at large in a long-term goal; and strategic context is highly justified by the results from the high correlation coefficients of the statistical testing and hypothesis verification, which shows a statistical significance, as vividly outlined and demonstrated with the proposed relationships that exist between brand, CSR and brand reputation.

Discourse as highlighted earlier remains dominant since its enactment in the Regan era.

Gardner et al (2003) mentioned: ‘Discourses reflect the general and enduring systems of thought which in subsequent influence the formation and expression or passage of ideas within a historically confined time.

Friedman argued that focus on social responsibility is a fundamentally subversive background and ideology in a free society.

Some schools of thought argued that profit-making organizations bear no legitimate interests in philanthropy; doing so is a distraction from their primary-key obligations and goals of making profits (Seeger and Hipfel 2007).

CSR can play a huge role in enhancing and promoting the brand; the consumers’ motivation for CSR and their perception of corporate brands (Mody et al. 2017a, b), and consumer satisfaction as recognized by Yang et al (2017) are important factors affecting consumer loyalty (Yang and Yin 2019).

Lu et al. (2020) pointed out and emphasized brand loyalty as a vital creation for the position of firms in the marketplace as well as a tool for creating competitive advantage.

Carroll (1979, 2008) presented the roles of organizations along the pyramid frame; constituting “legal, ethical, economic”, and a fourth discretionary leg.

In line with research findings (He and Lai 2014; Arslan et al. 2014), it was revealed that ethical and philanthropic practices have positive effects on brand images.

While Luo and Lv (2019) mentioned charitable donations as CSR; in this light, bearing and assertion organizations can go beyond this and imbibe ‘investment risks as a veritable tool as enumerated in this study.

Balancing environmental and social concerns with economic concerns rarely occurs and is evident in the way Carroll (1991) described CSR’s four segments.

The triple bottom line in its prominent or dominant place and significant position itself is all-encompassing and an encapsulating shell of societal marketing; as illustrated in Fig. 1 above, drawn from this study recently done by the author and proposed here; CSR can be carved and extrapolated from the triple bottom line.

Pertinently, as mentioned earlier, the statistical significance shown based on the *p* value (< 0.0001) from Table 13 is an indication that there is a strong connection between investment risks and culture, CSR, and brand reputation following the high R Sq. – value observed and seen from Table 12, and also from results of the correlation matrices from Tables 19 and 20 based on the relatively high values, and correlation coefficients as displayed. This supports the proposed model and basis for this research from Figs. 3, 4 and 6.

These findings align and are consistent with the outlined studies and literature (Mody et al. 2017a, b; Yang et al. 2017; Yang and Lin 2019) expressing consumers’ motivations, and brand attachment, which ties with culture.

### Emerging realities and pragmatism: managing risks

Climate change, mitigation, investment risks, models, projections, and litigation

#### Managing risks

Borne out of emerging realities, risks, and climate change challenges, consequences and effects it becomes crucial and exigent to look vividly into climate change mitigation and actionable steps or plans, risks associated and linked with climate change-related activities and management.

#### Climate change, mitigation, models, projections and litigation

Climate change was positively linked to electricity consumption and is projected to increase along with changing climatic variability and extreme or severe weather conditions (Craig 2016; McFarland 2015).

Due to electricity generation from fossil fuels for industrial uses, individuals and organizations; carbon emissions are continually being released and trapped in the Earth’s heat blanket or trapper within the atmosphere.

The carbon being trapped in the atmosphere as a blanket cover prevents solar radiation from passing through to be reflected back out of the atmosphere due to transparency loss.

The effect subsequently is this trapped solar heat radiation causes a number of environmental issues and hazards from global warming and entire climate change comprising; ‘polar ice–cap melting, flooding, rise in ocean tides, surges, precipitation increases, the intensity rises and perennial floods, food shortage from transparent water loss in plants, and acute water supply to plants nourishments, among several others, hazardously linked—related cause effects.

These obviously connect with human activities, business, and economic strives, motives or gains.

As revealed and unveiled by Heede (2014) and IPCC (2014); the majority of carbon emission's negative influences on global settings and frequently rising temperatures are attributable and connected to large energy-producing organizations.

Scientists have warned if no drastic measures, actions, and steps are taken to abate this trend seen; the consequences for humanity and the human race can be severely dangerous and catastrophic if the global temperature is not kept below or within 2 °C.

This has triggered several debates, arguments, and discourse among panels across nations, governments, stakeholders, policy – decision makings, political circus, and action groups.

Invariably some groups, entities and volunteers have actively woken up to challenge organizations that disrespect or abuse climate and environment, governments and bringing a significant rise and increases to pending lawsuits, litigation, and cases for jury hearings, declaration and proclamation in courts, and courtyards with some cases pending, on hold and adjourned.

#### Climate change: combating climate changes, stabilization, and mitigation efforts!

This cannot be left isolated nor dissociated in the subject and field of “sustainable marketing, business environment, and environment or environmental protection” as a consequence of the pre and modern industrial revolution eras and economic activities that have culminated to drastic changes in the climate and ambient conditions following various phenomena comprising; “carbon cycle changes, emissions, carbon-dioxide, greenhouse gas emissions, carbon sequestrations, arctic temperature variations and polar ice cap melting, pollution, even uncontrolled and unregulated nuclear emission”, sources, etc., could be extremely catastrophic.

In reference, the extension and continuation of the United Nations Frame for Climate Change (UNFCC) 2009 Copenhagen-s agreement; 2015 continued debate agreement established and re-iterated aiming for even 1.5 °C to protect island states more prone, threatened, and exposed to rising sea levels.

Furthermore, to expand and delineate this concept and phenomenon more vividly it is necessary to examine some models and predictive tools that caption and extrapolate into future gas emission trends, while cognizant of the roles of litigations as well, and looking beyond the climatic models.

For about and around the past two previous decades, efforts to mitigate emissions of carbon dioxide and other greenhouse gases have centered around, focused, and frequently driven around the goals of stabilizing atmospheric concentrations of these gases.

Efforts to mitigate carbon emissions, majorly carbon dioxide and other greenhouse gases have been frequently debated, keenly and intensely focused on the goal of stabilizing atmospheric concentrations of these gases.

This focus on atmospheric stabilization towards equilibration is historically rooted and grounded in the text of Article 2 of the United Nations Framework Convention on Climate Change (UNFCCC), in which is written:

*The ultimate or prime objective and prima aim of this Convention … is to achieve … stabilization of greenhouse gas concentrations in the atmosphere at a level that would prevent dangerous anthropogenic or man-made interference with the climate system and manifestations that could be seen.*

*(UNFCCC (UN Report,* 1992*)*

Towards this goal and aim or agenda, a considerable body of literature has evolved and emerged to attempt to first quantify what could be considered to be a ‘dangerous’ level of climate change, and second to determine what levels of greenhouse gas stabilization and equilibrium levels are consistent with avoiding said climate changes (Schneider and Mastrandrea 2005; Smith et al. 2009; Knutti and Hegerl 2008) and anticipated severities or unforeseen.

### Where are we?

Several templates and a vastly high number or amounts of assessments have ultimately considered how atmospheric GHG concentrations could be stabilized or equilibrated (Fisher et al. 2007) and forestall adverse build-ups and accumulations or sequestration while striving and strengthening efforts to establish equilibrium as a vital and key priority.

The lower the desired stabilization level, the sooner and faster global GHG emissions must peak and decline (Fisher et al. 2007) or subside. GHG concentrations are unlikely to stabilize and equilibrate this century or anytime sooner without major key policy changes (Rogner et al. 2007) or instruments^1^, and aggressive policy measures or steps by “stakeholders, parties and concerned” entities.

#### Projections and references

**N.B**:

This model was carved and generated based on an empirical equation following salient and relevant parameters fitted as produced and applied by the author toward future projections and predictions!

In a close and closer observation ‘predicting models can serve as veritable and potential tools giving warning signs and alert of possible global greenhouse and carbon rising levels while also enabling mitigating plans and proper actions towards averting potential disasters imminent from climate change events and as a strategic guide for corporations in drafting suitable CSR templates & mitigating plans.

Predicting models and estimates of greenhouse gas emissions and carbon metric tons estimates in the present model as presented and based on the empirical equation shows an increasing trend over the years starting 1900 beyond 2010.

The empirical novel predicting equation cognizant of the obvious incidences and present fact that the ‘global carbon level is incessantly and constantly increasing as observed and recognized by the author predicts the carbon estimated emission and greenhouse gases starting from 1900 to 2010 and beyond as long as the estimate keeps increasing.

**Fig. 7:**
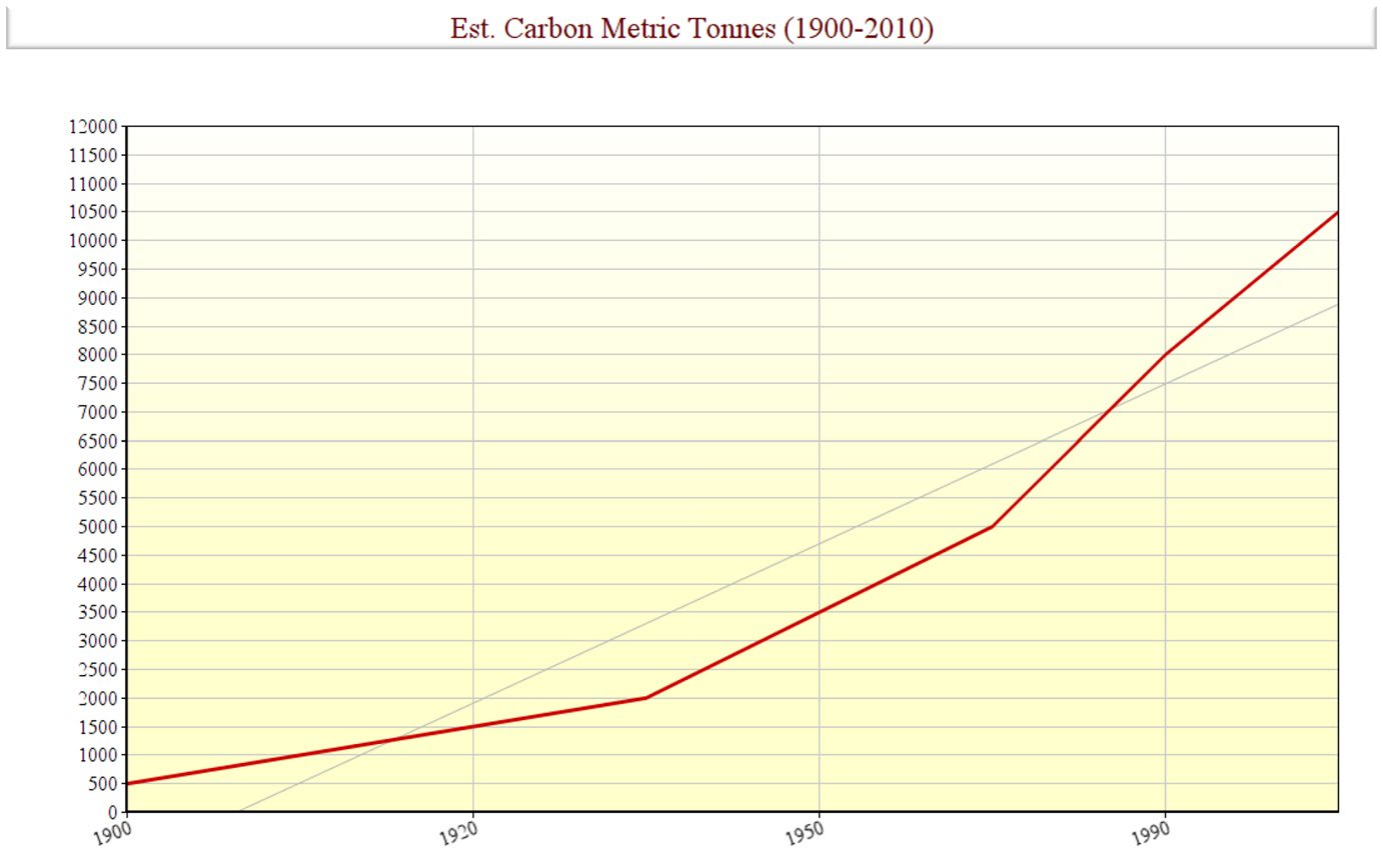
Estimate from our model starting 1900 beyond 2000. Reference and source citation: Adewole et al. (2018)

**Fig. 8:**
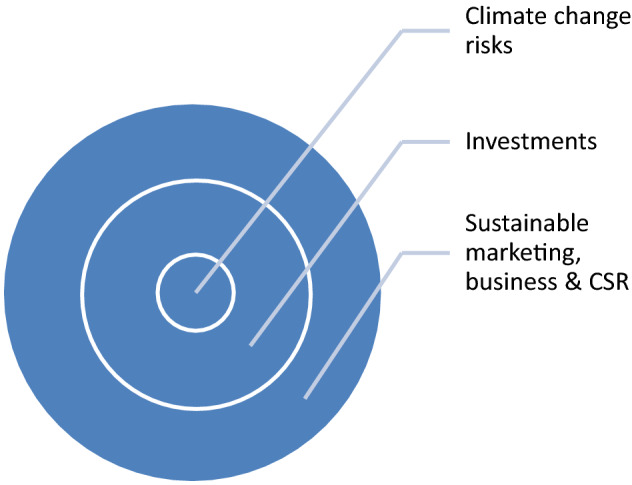
Proposed model of sustainable marketing incorporating investments and climate risks; Source: Present study draft and author’s draft

Comparison model (brief):

**N.B**:

The observed trend in the presented empirically carved and novel empirical model or equation based on linearized—exponential and media mobile as shown in Fig. 7 above is similar to trends observed as shown in the CO_2_ emissions in Fig. 8 as both show and exhibit increasing trends and found in literature documents.

The novel empirical model applied takes an exponential form of the type;

$$y=a.{b}^{x}.$$

#### Next perspective and future research direction

Revolving around this question earlier presented and put forward again:

Litigation, projections, CSR, pathway and climate change models, where are we?

This is the pertinent question that points to the next perspective and dictates the bearing of future research and investigation.

### Litigation, climate and dynamic model(s): 'Projections, realities and looking beyond!

Models available and formulated on climatic models can significantly help to predict and project the various gas emissions, exposures and obtain future estimates of atmospheric greenhouse gas emissions.

While mentioning and emphasizing the place and role of litigations, laws and enacts, acts or documentations in shaping policies and decision makings to climatic issues and related concepts; it is helpful seeing “models and litigation” as two complimentary indicators or assessment and evaluation parameters.

There is also an expedient need and advantage of looking beyond the models and present scenario while extrapolating and projecting into the future events or anticipated scenes to come and emerge!

#### Cases in emerging trends and legal selected reviews

Climate change litigation continues to expand across jurisdictions as a tool, check and veritable weapon or viable measures to and options strengthen climate action. Despite this direction or waves, evidence on the impacts of climate change litigation is still mostly found anecdotal (Setzer and Vanhala 2019) and seems not realistically true and reliable enough.

#### Increasing mitigation ambition—example cases

Quite prominent; “Urgenda Foundation vs. State of the Netherlands is the first case to argue successfully for the adoption of stricter emissions reduction targets, measures, and compliance by a government”.

The Court of Appeal of Hague on October 2018 rejected all the Dutch Government’s objections, including that the 2015 District Court decision infringed upon the principle of the balance of powers, while it upheld and ratified that the Government must reduce or cut emissions by at least 25 percent on 1990 levels by 2020^2^.

Following this development and its remarkably overwhelming success as the first ever of such cases to be won in lawsuit against the government, Urgenda, as an emerging precedent has invariably led and resulted in a number of similar calls and agitations calling for court actions and litigation, have recently emerged^3^.

#### Lower equity risk premium, risk aversion, and reputation management

Corporations can face severe economic damages when their corporate reputations and brands are assailed and badly painted or sales are affected by consumer boycotts. As contentiously argued by some rating agencies, a comprehensive CSR program package or outline will lower and considerably reduce a company’s equity risk premium. A direct correlation and co-existence between reputation and financial outcome measures—share price and credit rating (Hancock 2005) has been illustrated and presented through a model designed and carved by the global public relations company Bell Pottinger. In fact, companies may face a variety of legal and reputational risks if they do not have adequate social compliance or corporate social responsibility/sustainability programs in place.

#### Incorporating investment risks

As earlier stated and enumerated or pointed out; “marketing practices, business and economic activities” cannot be dislocated or isolated from climate changes, impacts, and mitigations.

In as much as climate change calls for mitigation following the drastic impact brought by business and economic activities on the environment coupled with the recent marketing practices; we cannot overemphasize the exigency and needs calling for drastic and meaningful actions and steps towards “pragmatism and practically realistic steps, actions and moves”.

Finally, and conclusively the sustainable marketing and business model would be the one all-encompassing in the wake of the present realities that incorporates “investment and climate change risks, adaptable, quite flexible and adjustable to meet present needs, not at the detriments of the environment and conscious of the future scenario and the future generations”.

In the real light, past scenes and events seen and recent trends in marketing, business, and economic impacts on the environment, climate, and society, sustainable marketing, practically realistic, is essentially crucial towards pragmatism to bring about mitigation where sought in particular on climate and abating the situation or challenging circumstances.

This way, if corporations are conscious enough, sensitive, and cautious of their actions and make concertedly practically realistic efforts to reduce impacts on the climate embracing sustainability as a watch tool and imbibe investments and climate risks we can meet the present needs of time while also pertinently preserving the planet and protecting the future and the interest and well-being of future generations.

The corporations can also conserve and save resources that goes into litigation processes against file suits and jury pending court cases as well which can be channeled towards better utilization of resources in meeting the social needs of the community and society while also enhancing and boosting their revenue streams, investment returns and enhancing profitability.

### Propositions and framework for hypothesis

#### Proposition and model: incorporating investments and climate change risks

As earlier mentioned and subsequently I am expanding on this in view of value creation and creating shared value in line with presentations by Porter and Kramer (2011) who presented the “Creating Shared Value (CSV) framework linking strategic CSR and sustainable outcomes” to a deeper appreciation of societal needs and a better understanding of the true bases of company productivity as given and explicitly presented.

This appears a good bearing and reference to pursue climate change and investment risks by ‘new path strategic—focus shift and positioning; the essence of sustainable business is to pursue value creation and reflect on this ideology, bearing and perspectives in every facet and aspect of its strategies, steps, daily, routine activities and operations.

Figure 8 as shown above and presented incorporates and vividly captures the investment risks in the climate novel climate model as proposed and presented.

Furthermore; it was established in 2002 at the ‘World’s Summit on ‘Sustainable Development, while the corporate leaders present—in unanimously agreed and articulated that businesses must constitute a major entity and participant in the sustainable program.

The growing interest in sustainability has been seen and grown over the past decade (Mcintosh 2007) in line with the literature.

Allen (2016) discusses a number of Multi-stakeholder engagements resulting in a significant number of global voluntary citizenship initiatives, p. 46 in terms of their bearing and importance to environmental sustainability among business, government, and civil society.

In line and deductions following the previous literature texts presentations review, extant working business environments on the large or macro scale, realities and emerging facts or occurrences seen so far till date and present, and following deductions and results of these findings on potential roles of the brand to climate mitigation as consumers advocate for brands connected or associated with CSR, in nexus and tied to culture within the working template of this work from Fig. 4; I am putting forward the propositions discussed below.

I'm proposing two feasible and pragmatic models and Perspectives within and potential response to the extant and present realities seen and emerging trends in marketing today and recent occurrences;

1. Incorporating investment and climate risks
2. Adopting a sustainable marketing frame and template flexibly capable of adapting to the present realities and abating the situation while rescuing, preserving, and protecting the future!

A sustainable marketing plan or frame which is adaptable and flexible and proactive would always be sensitive to the immediate or present realities of the day and flexibly adjust to meet the present needs while also being conscious and protecting future generations.

In view of the risen incidences of the vast number of action suits pending suits or court cases and jury cases against corporations, governments, and organizations; incorporating climate risks into the investment plan and templates while seeking alternative greener energy sources and non-fossil zero carbon emission sources as evident from this study could significantly 'abate the situation helping corporations to be on feet, more conscious of the actions they take impactful on climate or environment and cautious enough while able to save resources going legal cases and filings brought by plaintiffs in lieu of climate change protests.

Finally, I coined the word: “**SCRIR**: Social Corporate Responsibility and Investment Risks”, like insurance and ‘hedge; this can be imbibed and adopted into the CSR strategy of an organization within a cultural context extension as well which would potentially help in abating the climate changes protests and litigations appearing on the rising incidences over the past few years and recent events seen.

In essence; “value-creation and shared-value” constitute significant and vital key in CSR, marketing and business model implementation strategically.

In addition, and sequel to deductions and inferences drawn from a previous study report capturing the brand concept into the CSR ideology can enhance and promote a strong brand, this in my opinion can boosts value creation.

A socially responsible organization, firm or business is one that cares about the values and quality of orders delivered to its customers and consumers and is an entity or operating unit that cherishes and appreciates value creation, giving value to its customers and avoiding as well all risky and environmentally unfriendly practice sand awful practices.

Adapting and carving CSR models into business should emphasize value-based creation that turns products that give maximum satisfaction anticipated by customers or consumers and also environmentally friendly as a potential tool for abating the present situation and realities seen and the rising prominences and incidences in litigation, jury and pending legal cases for actions or redresses; obviously doing so by adapting and carving CSR models applied in business by corporations and by way of incorporation of investments and climate change risks; corporations can potentially enhance the revenue streams, re-channel resources utilization optimally and enhance their profits or margins.

#### Next perspective and future research direction

Revolving around this question earlier presented and put forward again:

**Sub-theme constitutive for the actionable plan, perspective next and directions**: litigation, projections, CSR, pathway, and climate change models, where are we?

### Litigation, climate and dynamic model(s): projections and looking beyond!

Models available and formulated on climatic models can significantly help to predict and project the various gas emissions, exposures and obtain future estimates of atmospheric greenhouse gas emissions.

While mentioning and emphasizing the place and role of litigations, laws and enacts, acts or documentations in shaping policies and decision makings to climatic issues and related concepts; it is helpful seeing “models and litigation” as two complimentary indicators or assessment and evaluation parameters.

There is also an expedient need and advantage of looking beyond the models and present scenario while extrapolating and projecting into the future events or anticipated scenes to come and emerge!

#### Cases in emerging trends, realities—pragmatism and legal selected reviews

Climate change litigation continues to expand across jurisdictions as a tool, check, and veritable weapon or viable measures to and options in strengthening climate action. Despite this fact and stemming towards the path of this direction or waves, evidence on the impacts of climate change litigation is still mostly found anecdotal (Setzer and Vanhala 2019) and seems not realistically true and reliable enough. This, obviously calls for more pragmatic steps, actions, and proactive efforts enough to save corporations of their resources spending spuriously wasteful pursuing litigation charges brought against them which led to the recommendations below.

**Major limitations/constraints**

One major constraint was fund limitation and lean resources in carrying out an intensive survey and study like this, which was single-handedly sponsored by the author with no external source of funding nor support.

There were also some language constraints, few communication barriers at the initial stage, and some cultural barriers, this was, however, overcome with some efforts by a few generous individuals on the streets who put some drinks and refreshments for me and some language interpretation by native friends.

Another severe constraint or limitation encountered was the pandemic situation making questionnaire administration and data capture more difficult, intense, and a bit harsh or severe as the 2nd phase of the field survey and poll was done in the pandemic peak and COVID-19 lockdown period of 2020.

**Implications of study findings to practitioners:**

- This research and findings will benefit practitioners and industrial in grasping the idea of doing business responsibly emphasizing steps and applications in avoiding legal suits and litigation cases arising from negligent practices resulting to climate change and hence saving and conserving resources wasted in reverting litigation suits brought against organizations.

It is essential to look extensively beyond prediction models while establishing and building on a brand and its relationship with CSR, while establishing in a strategic context of a practically realistic business model how this relationship can be translated to value-creation and applied in abating climate change, addressing all environmental concerns, redressing litigation incidences, which has been on the rise, among other issues or concerns resulting from impacts of business and socio-economic pursuits of humans.

Investment risks and a culture imbibing such forms a potential and veritable tool for checking corporations against climate change impacts and actions or activities corporations do that could adversely impact the climate and environment.

Conclusively; the sustainable marketing and business model would be all-encompassing in the wake of the present realities incorporating “investment and climate change risks, adaptable, quite flexible and adjustable to meet present needs; environmentally non-detrimental and conscious of the future scenario and the future generations”.

#### Recommendations

- Adapting and carving CSR models into business would enable and foster value-based creation that turns products that give maximum satisfaction anticipated by customers or consumers and also environmentally friendly while fostering cultural and family-relational building or nexus and bound as a potential tool for abating the present situation and realities seen and the rising prominences and incidences in litigation, jury and pending legal cases, thus corporation should imbibe this steps as outlined.
- In light of the above findings and presentations; ‘Incorporating investment and climate risks and adopting a sustainable marketing frame and template flexibly capable of adapting to the present realities and abating the situation while rescuing, preserving and protecting the future!
- Corporations are urged and suggested to consider critically and in-depth their strategies and modules in the extension of CSR to incorporate investments and climate change risks as this potentially highly promises to abate the current situation, realities seen and lessen rising incidence profiles of jury law cases and suits for legal action calls and redress towards better, optimal and more or higher efficient channeling to the utilization of resources and bearing on revenue streams, gains, and profitability in capture or returns.

These recommendations are buttressed and strongly supported in line with the results and findings of the hypothesis testing presented and the result of the ANOVA statistics summary.

As indicated by the *p*-value obtained (> 0.05) in the ANOVA testing correlation matrix scheme or Tables from 16, 17 and 18, then subsequently in 19 & 20, and the hypotheses frames, thus, it is enough to empirically arrive at the deduction and conclusions made above that investment risks should be drafted into the organization’s strategy and embedded as a& key and salient aspect or core - part of the corporate culture.

## Conclusion

Investment risks if embraced and adopted as a culture together with building a strong relational brand via CSR at the corporate level can do a lot of help in helping organizations achieve resources optimization and uses while avoiding unnecessary wasteful spending in seeking legal redress and trying to overturn cases and suits brought against their negligent actions towards climate changes and environmental damages.

More pertinently these resources rather than go into legal spending against litigations and seeking the favor of rulings in court against them brought by plaintiffs can better be channeled into social spending more responsibly to improve and make a better living and enhanced quality of life for the community and localities in which they operate in meeting their social needs.

Interestingly; referring to a study done by Lu et al. (2020), it was emphasized consumers do in fact recognize what CSR is. The majority of consumers know what CSR is and how important to firms (Lu et al. 2020); the main outcome of their research is that CSR initiatives have positive effects on brand loyalty and brand image in line findings of this research that a relationship and connection exists between CSR and brand relationship and brand positioning thus showing why companies should do more and carve into their models and strategies.

Adapting and carving CSR models into business should emphasize value-based creation that turns products that give maximum satisfaction anticipated by customers or consumers and also environmentally friendly as a potential tool for abating the present situation and realities seen and the rising prominences and incidences in litigation, jury, and pending legal cases.

While the climate change models and predictive tools could help gain possible insights and grasp ideas into future trends, we have to look beyond the models, embracing more pragmatic and realistic actions and tools more responsible, by imbibing, and strategically adopting investment risk culture tied and building around the corporate brands and strongly emphasize corporate social responsibility and other tools among; “stakeholder’s engagement, social contract theory and societal marketing” as previously elaborated and explained.

The results and statistics seen in this study justify the present models and novel formulations; the high *R*-squared values seen in the tables such as a high value of 0.9753 justifies the fact that investment risks as a culture would enhance a strong brand reputation as it can be inferred that consumers would likely associate with brands that care for the environment and conscious of the impacts of their activities on the environment, consumers and society at large. The significance of the brand and as an instance, in its translation to equity, also in driving consumers’ motivation is clear, and evident as revealed (Mahmood and Bashir 2020). This is also consistent with other literature revelations as evident (Dawood 2019; Irsbad et al. 2017; Lu et al. 2019; Lins et al. 2017).

As indicated by the *p*-value obtained (> 0.05) in the ANOVA testing, correlation matrix scheme or tables in Figs. 3.7–3.7b), and the hypotheses frames as well it is enough to empirically arrive at the deduction and conclusions made above that investment risks should be drafted into the organization’s strategy and embedded as a key and salient aspect or core-part of the corporate culture.

From obvious facts seen in this investigation and observations as revelations emanating and built on present study findings drawing salient and relevant insights making extrapolations:

Inferring from the test 1 hypothesis verification and based on this evidence earlier affirmed statistically there is a relationship between CSR and the brand image of an organization. In connection and bearing with test hypothesis 2 CSR would enhance the brand image of an organization, image, and quality or brand equity and supported by the emerging model presented and illustrated in Fig. 4 above, also consistent with literature among; Mahmood and Bashir (2020), and subsequently expanded upon in Fig. 6 showing explicitly the brand connections among highlighted mix(s) and furthermore in Fig. 5 highlighting its implications incorporating investment risks by adopting a value-based marketing and socially responsible or societal marketing orientations.

While looking at the present issues associated with the business environment of environmental concerns on how the business could impact the environment and not overlooking the rising trends of jury cases and pending proceedings in court calling for actions by way of legal redress against corporations and governments brought and filed by individuals to action movement groups, I strongly sought and anticipate to look at the twenty-first century perspective of doing business in a sustainable manner and towards achieving a dream twenty-first-century greener safer planet.

The poll findings emanating from this study showing relatively high proportions up to 96.0% as observed and revealed by responses on the public side shows and indicates the rising litigation incidences ‘prompting for redress and compensations is not unconnected with the high demand of investment risks as part of corporate structure, culture and brands.

CSR as a strategic tool has been extensively outlined while enumerating stakeholder’s perspectives (Heikkurinen 2018), it goes a long way as well as a strategic instrument in resource utilization (Potter and Kramer 2006).

While climatic models and predicting tools are essential, potentially and extremely useful as predicting tools and insights indicate that we have to look beyond the models and strike the right balance in doing business sustainably, climate change, litigations, and mitigations or steps achieving such.

More at large CSR can impact and reflect on how companies shape and adapt their strategies, brands, and business models around climate change mitigation efforts, addressing environmentally related issues from impacts and activities of companies and corporations, checking their negligent practices, engendering a potential trend reverser to rising incidences of litigations, jury cases and activism against states from actions and activities bringing concerns and serious impacts or effects on climate and the environment while working a practical and realistic path towards sustainability and building brand relationship.

Importantly; the sustainable marketing and business model would be the one all-encompassing in the recent wake of the present realities that incorporates “investment and climate change risks, adaptable, quite flexible and adjustable to meet present needs not at the detriments of the environment and conscious of the future scenario and the future generations” while promising high hope and greening for a safer and preserved planet.

## Supplementary Information

Below is the link to the electronic supplementary material.

Supplementary file1 (DOCX 17 KB)

## Data Availability

The data applied and used in this research work were from questionnaire administration and personal interview conducted by the author in the course of this research project and investigation. The data was compiled and available with the author on request.

## Footnotes

1 Shanks and Roegner (2007) Aquatic Climate Change Adaptation Services Program-Pacific……..publications.gc.ca>collection_2016>mpo-df0>Fs97-4-3049-eng

2 www.urgenda.nl.

3 https://www.ejiltalk.org/a-new-classic change in climate-change-litigation: the –dutch-supreme court-decision—in-the-urgenda-case/.
