# Supplementary material for: CSR–brand relationship, brand positioning, and investment risks driven towards climate change mitigation and next perspectives emerging from: “Litigation, projections, pathway, and models”
Source: SN Bus Econ. 2022 Dec 20;3(1):18. doi: 10.1007/s43546-022-00374-4 (PMC9763811; doi:10.1007/s43546-022-00374-4)
Supplement: Supplementary file 1 — Supplementary file1 (DOCX 17 KB) [file 43546_2022_374_MOESM1_ESM.docx]

**Appendix**;

‘

In line with the research question and the ‘hypothesis drawn in line with

investment risks as a key component to be embedded in the brand strategy and ‘CSR; a relatively high likelihood of most members of the public domain seeing this perspective in strong co – relation, bearing and agreement with the top management team position and employee below the management cadre.

‘*

The relatively high mean justified this!

‘**APPENDIX II**:

‘*

‘

Draft questionnaire administered;

**References frame:**

**‘**

**‘***

2) What is the role and potentials of the brand and ‘CSR in mitigating climate changes risks?

3) How do individuals and entities within a corporate organization perceive the brand?

**‘**

**Hypothesis:**

1. By incorporating investment risks and climate change risks; corporations can achieve optimal utilization of resources.
2. Investment risks should be seen and perceived as a culture, style and manner embedded into the ‘CSR strategy of an organization.

In reference and line with the earlier proposed questions and ‘hypothesis frame formulation:

‘The following research questions were drafted for the field poll and opinion capture:

‘*

Management teams, employee cadre & public domains:

Is investment risk important and do you consider it key in achieving ‘optimal resources conservation and utilization in your organization?

‘

Conceiving ‘CSR as a brand and tied to brand relationship is this in connection with investment risk a right pragmatic step as a tool and structure or device for climate change mitigation?

To what extent do you agree on the previous position?

1. strongly b). strong c), mild d). less e). not sure or varies

‘

Do you conceive or perceive the brand as a culture and link or tie with the investment risk?

To what level and extent do you perceive the ties between brand and investment risk:

a) strongly b). strong c). mild d). less e). not sure or varies

‘

‘Will achieving optimal resources utilization embracing ‘CSR & investment risks into the brand be quite beneficial and if channeled as corporate philanthropic spending and charity?

‘**Public segment**:

‘

Are you a proponent for seeking legal redress against organizations for ‘climate change actions?

To what extent or degree do you agree on this position as a proponent in seeking legal redress against climate change actions?

a). strongly b). strong c). mild or d) less e). not sure or varies

‘

;

Do you feel ‘investment risks should be harnessed into compensations for redress against climate change actions and litigation suits filed against corporations by the plaintiffs?

To what extent do agree with this position?

a). Strongly b). strong c). mild d). less e). depends

‘

Have you sought or engaged in a previous jury or legal cases brought against corporations for ‘climate change actions?

‘

What informed the basis of your decisions or position on investment risks as a imposition or check on corporations in climate change mitigation and redress from legal suits?

1. Compensation b), warnings or reprimand c). Balance or check d). Social justice e). Others

You may explain your choices or other positions as deemed!

…………………………;

…………………………………………..;

:
